# Cutaneous Electrohydraulic (CUTE) Wearable Devices for Pleasant Broad‐Bandwidth Haptic Cues

**DOI:** 10.1002/advs.202402461

**Published:** 2024-09-06

**Authors:** Natalia Sanchez‐Tamayo, Zachary Yoder, Philipp Rothemund, Giulia Ballardini, Christoph Keplinger, Katherine J. Kuchenbecker

**Affiliations:** ^1^ Haptic Intelligence Department Max Planck Institute for Intelligent Systems Heisenbergstr. 3 70569 Stuttgart Germany; ^2^ Robotic Materials Department Max Planck Institute for Intelligent Systems Heisenbergstr. 3 70569 Stuttgart Germany; ^3^ Institute for Control Engineering of Machine Tools and Manufacturing Units University of Stuttgart Seidenstraße 36 70174 Stuttgart Germany; ^4^ Paul M. Rady Department of Mechanical Engineering University of Colorado Boulder, 1111 Engineering Drive Boulder CO 80309 USA; ^5^ Materials Science and Engineering Program University of Colorado Boulder, 1111 Engineering Drive Boulder CO 80309 USA

**Keywords:** contact feedback, haptic feedback, HASEL actuators, soft robotics, wearable devices

## Abstract

By focusing on vibrations, current wearable haptic devices underutilize the skin's perceptual capabilities. Devices that provide richer haptic stimuli, including contact feedback and/or variable pressure, are typically heavy and bulky due to the underlying actuator technology and the low sensitivity of hairy skin, which covers most of the body. This article presents a system architecture for compact wearable devices that deliver salient and pleasant broad‐bandwidth haptic cues: Cutaneous Electrohydraulic (CUTE) devices combine a custom materials design for soft haptic electrohydraulic actuators that feature high stroke, high force, and electrical safety with a comfortable mounting strategy that places the actuator in a non‐contact resting position. A prototypical wrist‐wearable CUTE device produces rich tactile sensations by making and breaking contact with the skin (2.44 mm actuation stroke), applying high controllable forces (exceeding 2.3 N), and delivering vibrations at a wide range of amplitudes and frequencies (0–200 Hz). A perceptual study with 14 participants achieves 97.9% recognition accuracy across six diverse cues and verifies their pleasant and expressive feel. This system architecture for wearable devices gives unprecedented control over the haptic cues delivered to the skin, providing an elegant and discreet way to activate the user's sense of touch.

## Graphical abstract

This article introduces a system architecture that combines a custom materials design for soft electrohydraulic actuators with a comfortable mounting strategy that places the actuator in a non‐contact resting position to deliver salient and expressive haptic sensations with high stroke, force, and bandwidth. A perceptual study achieves 97.9% identification accuracy and proves the presented sensations to be remarkably pleasant.

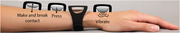

## 1 Introduction

The skin is the human body's largest organ and can perceive diverse tactile stimuli including soft contacts, pressure, high‐frequency vibrations (up to 1000 Hz^[^ ^1^, ^2^ ^]^), heat, and pain.^[^ ^3^ ^]^ Haptic feedback devices have great potential for sending meaningful information to users by employing the skin as a communication platform.^[^ ^4^ ^]^ Such technology can be used to enhance immersion in virtual and augmented reality,^[^ ^5^ ^]^ or to complement or replace audio and visual cues in loud or visually demanding scenarios.^[^ ^6^ ^]^

There are two types of skin with different tactile acuity.^[^ ^7^ ^]^ Glabrous skin (i.e., non‐hairy skin predominantly found on the lips, palms, and soles of the feet) is particularly sensitive and can detect small variations in applied forces and displacements (e.g., fingertip detection thresholds of 1.5 mN^[^ ^8^ ^]^ for a point force and 24 µm displacement for 20 Hz vibration^[^ ^9^ ^]^). Due to the high spatial and temporal acuity of glabrous skin, wearable haptic devices are often designed to deliver tactile stimuli directly to the fingerpad.^[^ ^10^, ^11^, ^12^ ^]^ However, such designs encumber the hands of the user, preventing free interaction with the environment and limiting the application space of such devices. Non‐glabrous, hairy skin covers the majority of the body surface (about 90%^[^ ^13^ ^]^) and plays a fundamental role in the perception of pleasant sensations^[^ ^14^ ^]^ due to the presence of C‐tactile afferents that respond to slow‐moving touch with low indentation forces (0.3–2.5 mN^[^ ^15^, ^16^ ^]^). This type of skin has a lower spatial density of mechanoreceptors, resulting in lower sensitivity compared to glabrous skin (e.g., forearm detection thresholds of 2.6 mN^[^ ^8^ ^]^ for a point force and 149 µm for 20 Hz vibration^[^ ^9^ ^]^). Locating haptic feedback devices on the hairy skin keeps the hands free and enables larger skin areas to be used for haptic communication, but the lower cutaneous sensitivity requires higher forces and displacements to generate perceivable stimuli,^[^ ^12^ ^]^ thereby rendering such devices more challenging to realize in a compact and comfortable form factor.

Both types of skin can perceive a wide variety of mechanical stimuli, such as broad‐bandwidth vibrations, the start and end of contact, sustained pressure, shear forces, and complex combined stimuli.^[^ ^17^ ^]^ However, most haptic actuation systems cannot reproduce this range of tactile sensations. The most commonly used actuators for haptic feedback are eccentric rotating mass (ERM) motors and linear resonant actuators (LRAs), both of which provide only vibrations.^[^ ^12^, ^18^ ^]^ ERMs cannot independently vary the amplitude of vibration and frequency,^[^ ^19^ ^]^ while LRAs operate efficiently only near their resonant frequency, considerably limiting the range of sensations they can transmit. Moreover, these actuators typically produce vibrations at frequencies above 100 Hz^[^ ^10^ ^]^ that are very salient to both types of skin,^[^ ^20^ ^]^ thus making them particularly useful for attracting the user's attention. Yet, users often report that extended vibrotactile feedback is uncomfortable and annoying, sometimes even causing unpleasant tingling sensations or numbness.^[^ ^21^, ^22^ ^]^

An alternative method for producing salient haptic sensations is contact feedback, which involves physically making and breaking contact with the skin. New contacts capture the user's attention because changing tactile stimuli are more noticeable than constant stimuli due to sensory adaptation.^[^ ^23^ ^]^ The sensations of making and breaking contact are common in many physical interactions and have been found to provide strong and appealing sensations^[^ ^24^ ^]^ that are more effective at guiding user motion than vibrating, squeezing, or twisting the skin.^[^ ^25^ ^]^ Yet, this type of haptic feedback is underutilized and can be difficult to achieve because it requires actuators that produce large and controllable displacements in combination with suitable grounding of reaction forces. Thus, wearable haptic devices usually maintain contact with the skin and cannot provide contact feedback.

Voice‐coil actuators are a potential solution for generating versatile haptic cues since they can deliver sustained pressure in addition to vibrotactile feedback.^[^ ^12^ ^]^ However, voice‐coil actuators continuously draw current when holding an output force and generate heat during operation, resulting in reduced efficiency^[^ ^26^ ^]^ and potential burn risks^[^ ^27^ ^]^; furthermore, their mechanical design often results in large, rigid devices.^[^ ^28^, ^29^, ^30^ ^]^ More generally, simultaneously providing different haptic stimuli (such as vibration, contact feedback, and variable pressure) on the hairy skin typically results in heavy and bulky devices (e.g., refs. [25, 28, 31, 32, 33]) due to the lower sensitivity of this type of skin and the underlying limitations in traditional electromagnetic actuator technology.

Haptic devices with small form factors and large displacements can be achieved through the use of pneumatic actuators, featuring steady‐state output and low‐frequency vibration in conformal packages that can adapt to the skin.^[^ ^34^, ^35^, ^36^, ^37^ ^]^ Larger wrist‐wearable pneumatic devices can press into the skin with forces over 10 N.^[^ ^38^, ^39^ ^]^ Low‐frequency actuation (<20 Hz) has been found to convey calming, relaxing, and pleasant sensations, as well as intuitive concepts such as aliveness (e.g., bubbles at about 2 Hz, the human respiratory rate of about 0.8 Hz.^[^ ^40^ ^]^). However, pneumatic devices typically have a long response time, which adds a delay to real‐time haptic feedback and limits their operation at high frequencies (typically driven below 50 Hz).^[^ ^41^ ^]^ Additionally, most require bulky air tubes and/or must be tethered to heavy, noisy, and inefficient air compressors, limiting their application space.^[^ ^42^, ^43^ ^]^

In contrast to pneumatic actuators, soft electrostatic actuators can be driven by compact, efficient, electronic power supplies.^[^ ^44^, ^45^, ^46^, ^47^ ^]^ Dielectric elastomer actuators (DEAs) have been used to render static pressure and high‐frequency vibration,^[^ ^48^, ^49^, ^50^, ^51^ ^]^ but they are prone to electrical breakdown and require stretchable materials that present manufacturing challenges.^[^ ^52^ ^]^ Hydraulically amplified self‐healing electrostatic (HASEL) actuators couple electrostatic actuation with hydraulic pressure^[^ ^53^, ^54^, ^55^, ^56^ ^]^ and show strong potential for driving haptic devices due to their tunable forces and displacements,^[^ ^57^ ^]^ quiet and controllable soft actuation,^[^ ^58^ ^]^ low power consumption,^[^ ^59^, ^60^ ^]^ and actuation frequencies ranging from 0 Hz to beyond 100 Hz.^[^ ^53^, ^61^ ^]^ However, HASEL actuators typically employ exposed electrodes with high voltages (>1000 V) and therefore require additional materials innovations to ensure safe contact with the skin while maintaining reliable high‐performance operation at small scales. Additionally, devices and system designs that enable high‐frequency operation, contact feedback, and suitable mechanical grounding of actuator reaction forces are needed to deliver diverse cutaneous cues.

Hydraulically amplified taxels (HAXELs)^[^ ^62^ ^]^ are a type of electrohydraulic actuator that builds upon HASEL technology and is specialized for tactile feedback. HAXELs consist of slim electrically insulated actuators (6 × 6 × 0.8 mm) that drive a central 2.5‐mm‐diameter bubble. They provide focused tactile feedback on the fingertips, outputting up to 0.5 mm of displacement and up to 0.25 N peak‐to‐peak force at 80 Hz. HAXELs deliver haptic cues with good recognition on the fingertips and can also output vibration and shear feedback in constant contact with the skin. However, even with larger designs (10 mm width) that provided increased forces (0.75 N), relatively low recognition rates were achieved when they were adhered to less‐sensitive parts of the body.^[^ ^63^ ^]^

**Figure 1:**
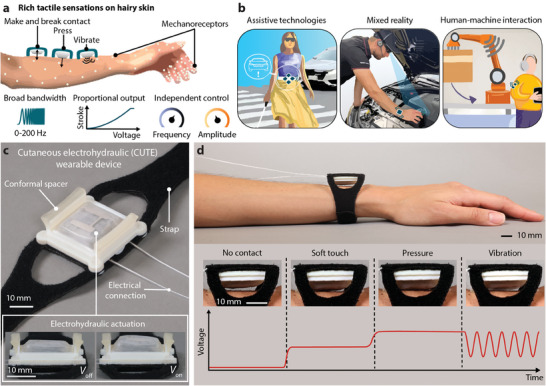
System architecture for cutaneous electrohydraulic (CUTE) wearable devices that harness electrohydraulic actuation to deliver rich and pleasant haptic sensations to the hairy skin. a) Our devices provide an expressive range of cutaneous cues on the user's hairy skin, including making and breaking contact, pressing, and vibrating with independently controllable frequency and amplitude over a wide bandwidth; the output stroke is proportional to the input voltage. b) Possible fields of application for wearable, compact, and expressive cutaneous devices. c) Prototypical CUTE wrist‐wearable device (37 × 40 × 15 mm plus strap) with an inset showing actuation in free space. d) The four main actuation modes of the wrist‐wearable device; note the details of contact between the electrohydraulic actuator and the user's skin.

Thus far, there are no wearable devices capable of delivering diverse and controllable cutaneous cues, including contact feedback, variable pressure, and broad‐bandwidth vibration, in a compact form factor and with sufficient force and displacement to be salient on the hairy skin. In this work, we present a system architecture for a new class of compact and expressive cutaneous devices that safely leverage electrohydraulic actuation for large stroke (2.44 mm), high force generation (2.3 N), and broad‐bandwidth (0–200 Hz) motion, thereby enabling the delivery of highly customizable, diverse haptic cues to hairy skin (**Figure** **1**a), resulting in discreet, localized haptic feedback for myriad applications (Figure 1b). Extending this concept to multi‐degree‐of‐freedom actuators or multi‐unit arrays^[^ ^47^ ^]^ will facilitate the output of more complex tactile sensations such as shear forces and spatial patterns, but here we focus first on carefully characterizing the underlying principles, capabilities, and user perception of a single degree of freedom (Figure 1c).

The high performance and safe operation of our system architecture are derived from a new materials design that includes low‐resistance electrodes, actuator encapsulation, and an elastomeric membrane that serves the dual purposes of electrically insulating the device and providing a restoring force to enable high‐bandwidth actuation. This materials design, coupled with a new actuator geometry, enables small‐footprint actuators that can be stacked to increase their stroke without substantial reduction in force, leading to compact and effective haptic devices. These wearables are quiet, consume low power, and do not heat up during operation. To allow contact feedback, we introduce a comfortable mounting strategy that places the electrohydraulic actuators in a non‐contact resting position and provides grounding of reaction forces for strong haptic sensations.

We demonstrate the effectiveness of this approach through the creation of a compact cutaneous electrohydraulic (CUTE) device capable of delivering distinct tactile cues to the dorsal side of the wrist (Figure 1c,d). In a perceptual study, fourteen adults achieved very high recognition accuracy (97.9%) over six haptic signals, including five active cues and one reference cue with no output. Participants then felt, described, and rated eleven CUTE haptic cues (including the first five) using the circumplex model for affect,^[^ ^64^ ^]^ which is a standard representation of emotion. The results show that our device is highly comfortable and creates recognizable haptic cues that communicate diverse sensations ranging from calming to exciting. Remarkably, users perceive almost all of these wide‐ranging cues to be pleasant; only the steady vibration was deemed unpleasant. Our prototypical cutaneous electrohydraulic device demonstrates salient, expressive, and pleasant haptic cue delivery, thereby validating the effectiveness of our presented system architecture and illustrating its potential for compact wearable devices that can deliver a wide range of cutaneous sensations.

## 2 Results

### 2.1 Materials Design for Safe‐to‐Touch, Small‐Footprint Electrohydraulic Actuators

**Figure 2:**
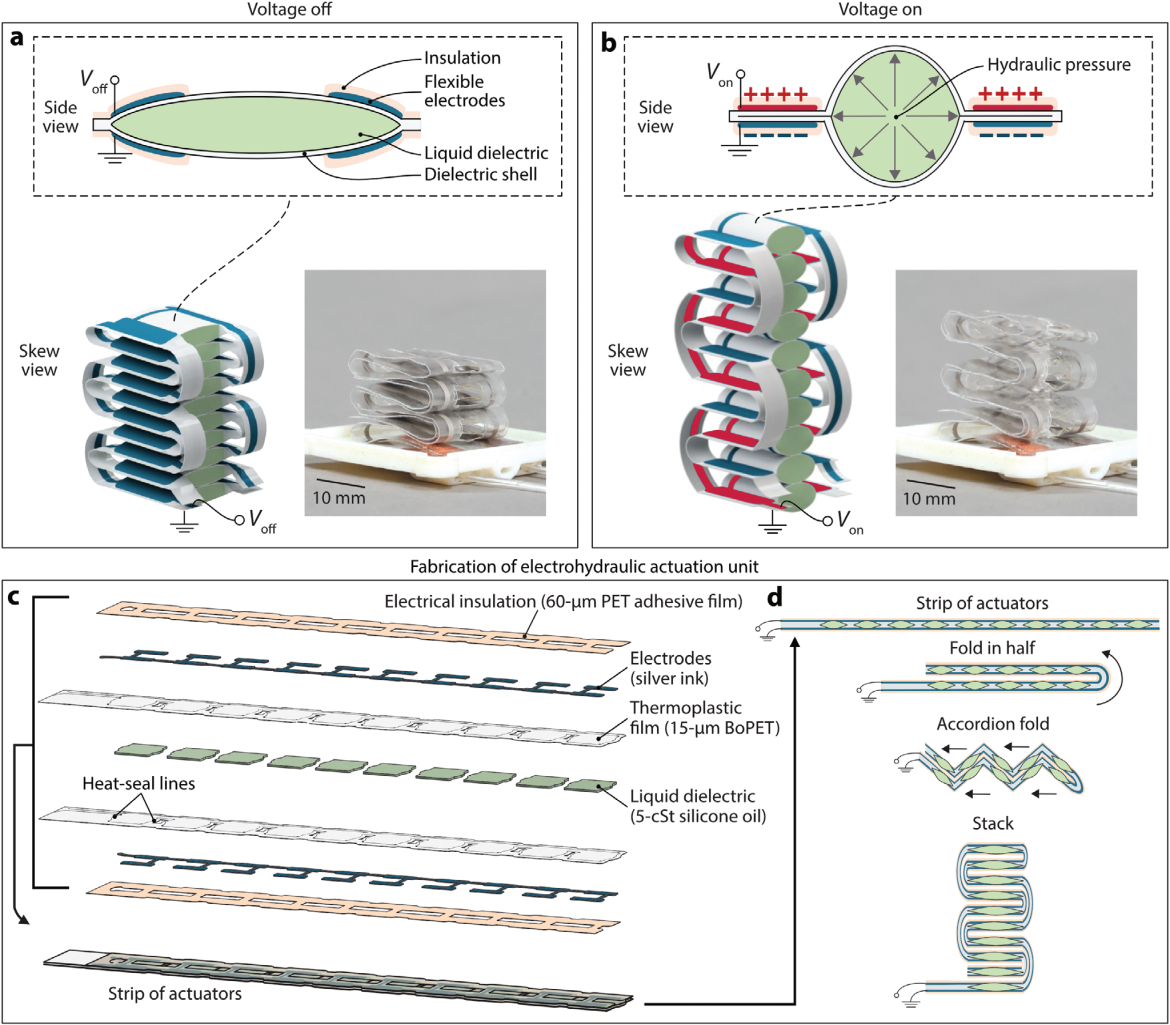
Materials design for safe‐to‐touch, small‐footprint CUTE actuators. a) An individual electrohydraulic actuator pouch consists of a flexible yet inextensible dielectric shell filled with liquid dielectric; the edge regions of both sides of the pouch are covered in flexible electrodes and insulation. A stack of ten pouches increases actuation stroke. b) Under sufficiently high voltage, the electrodes zip together, resulting in the expansion of the center region of each pouch in the stack. c) Multi‐material structure of the electrohydraulic actuator strip used to fabricate a stack of ten pouches. d) Folding of the assembled actuator strip to achieve an actuator stack where all exterior electrodes are grounded.

Our system architecture is driven by soft electrohydraulic zipping actuators based on HASEL technology.^[^ ^53^, ^56^ ^]^ Each actuator consists of a pouch made from a flexible, inextensible thermoplastic shell that has electrodes on both sides and is filled with liquid dielectric (**Figure** **2**a). When a voltage is applied across the electrodes, the electric field generates an electrostatic Maxwell stress that acts on the dielectric layers. A sufficiently high voltage causes the electrodes to begin zipping together and displace the liquid dielectric, thus increasing the hydraulic pressure within the pouch and resulting in an expansion of the part of the pouch not covered by electrodes (Figure 2b).

We use a square pouch geometry (14 × 14 mm): each pouch has two separate pairs of electrodes located in opposing edge regions that progressively zip toward the center. We compared this design to one with the same footprint but two neighboring pouches (similar to refs. [47, 65]); experiments demonstrated that the single‐pouch design provides higher forces and displacements (Figure S1, Supporting Information).

To fabricate the actuators, we heat‐seal a series of pouches from two sheets of 15‐µm‐thick BoPET film and screen‐print the electrode pattern with silver ink on both sides (Figure 2c), following the process described by Mitchell et al.^[^ ^66^ ^]^ Silver ink has lower resistance (<0.005 Ω sq^–1^ for 25 µm thickness) than the carbon ink (<50 Ω sq^–1^ for 25 µm thickness) used in many previous works^[^ ^54^, ^58^ ^]^; with the narrow electrode patterns needed for our small actuator stack, carbon electrodes heat up during high‐frequency actuation, while silver electrodes do not. We next apply an insulating layer over the electrodes; an adhesive 60‐µm‐thick PET film is laser‐cut in a prescribed pattern (Figure 2c) and applied over both sides of the strip of pouches, fully covering the electrodes but minimally covering the electrode‐free areas. We then fill the strip of actuators with 0.08 mL of 5‐cSt‐viscosity silicone oil per pouch.

The PET adhesive layer electrically insulates the electrodes, enabling safe operation of the actuators in close contact with human skin. Further, this layer prevents electrical arcing through the air between the high‐voltage electrode and the ground electrode. This insulation is the first of four safety mechanisms in our system architecture described in Section 2.3 and the Supporting Information. For typical HASEL actuators in prior work, about 10 mm of excess film had to be left at the edge of each exposed electrode to ensure an air gap of sufficient length to prevent this arcing, adding mechanical constraints that reduce actuator performance (especially at small scales) and increasing actuator footprint.^[^ ^57^ ^]^ Our added insulation layer allows us to omit nearly all of this excess film and is therefore key for enabling small‐footprint, high‐performance actuators.

The complete strip of actuator pouches is folded into a stack using a folding pattern that places the ground electrodes along the exterior (Figure 2d); we first fold the strip in half and then fold it accordion style. Further details on fabrication and the effects of the volume of liquid dielectric in each pouch are provided in the Experimental Section and in the Supplementary Materials and Methods, Figures S2 and S3 (Supporting Information).

### 2.2 Quasi‐Static Performance of the Electrohydraulic Actuation Unit

**Figure 3:**
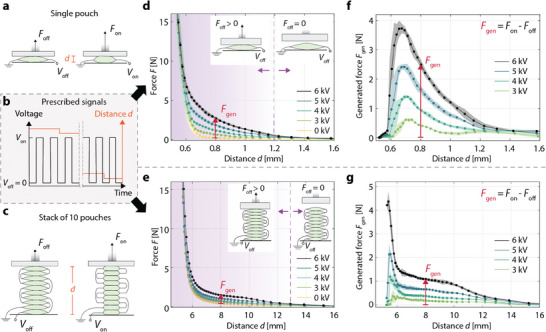
Characterization of actuation performance for a single pouch and a ten‐pouch stack. a,c) Schematics of the measurement using a dual‐mode muscle lever, a device that can prescribe a displacement or force profile while measuring force and displacement. b) The force was measured at a range of distances spanning the displacement range of the respective actuator, using five prescribed voltages (0, 3, 4, 5, 6 kV) at each distance. d,e) Mean measured force and standard deviation in force (n = 9, shaded region) for the single pouch and the ten‐pouch stack as a function of distance and voltage. f,g) Mean force and standard deviation (n = 9) generated by each actuator configuration across its distance and voltage ranges.

To characterize the force‐displacement curves and the generated forces of single‐pouch electrohydraulic actuators, we placed individual pouches at a range of distances *d* (measured from the base of the actuator) from a dual‐mode muscle lever and applied ramped square‐wave actuation voltages of 3, 4, 5, or 6 kV (**Figure** **3**a,b; Figure S4, Supporting Information). Stacks of ten pouches were characterized in the same way (Figure 3c). For both configurations (single pouch and ten‐pouch stack), we tested three actuator samples and processed three actuation cycles at each displacement. Figure 3d,e shows the mean measured forces and standard deviations (SDs) across the nine force measurements as a function of the distance *d* for both configurations. A force *F*_off_ > 0 N at a voltage of *V*_off_ = 0 kV indicates that the actuator was in contact with the muscle lever in the relaxed state, i.e., the actuator experienced a pre‐load.

To determine the force *F*_gen_ that is actively generated by the single‐pouch actuator and the ten‐pouch stack, we calculated the difference in force between its on and off states (Figure 3f,g). Single‐pouch actuators generated an average peak force of 3.7 ± 0.2 N (mean ± SD), while the ten‐pouch actuators generated an average peak force of 4.4 ± 0.4 N. The ten‐pouch actuators generated forces at substantially higher distances than the individual pouches (Figure 3f,g). This property of stacks facilitates their use in haptic devices that place the resting actuator out of contact with the skin, as it must have sufficient stroke to travel through the air gap and deliver touch sensations to the skin. Further, generating forces at higher distances is desirable for delivering cues to compliant human tissue, which deforms during contact.

### 2.3 Cutaneous Electrohydraulic Wearable Device

**Figure 4:**
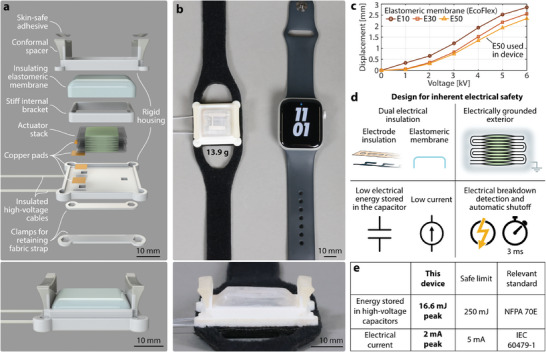
Cutaneous electrohydraulic wearable device. a) Components and structure of the CUTE wrist‐wearable device. b) The prototype has a footprint similar to a commercially available smartwatch (Apple Watch SE, 44 mm) and a mass of only 13.9 g. c) Device displacement when fitted with three different elastomeric membranes. d) Multi‐layer electrical safety design. e) Energy stored and electrical current in the actuator.

We propose a system architecture that leverages the proposed materials design to create wearable devices that deliver broad‐bandwidth haptic cues. We demonstrate the effectiveness of this approach through a prototypical CUTE wearable device, as shown in Figures 1c,d, and **4**, and Movie S1 (Supporting Information). This device is lightweight (13.9 g without cables) and has a compact and easily wearable form factor; it consists of a ten‐pouch electrohydraulic actuator covered with an elastomeric membrane and suspended off the skin in a rigid housing (Figure 4a,b). This mounting strategy provides effective mechanical grounding of reaction forces generated when the soft actuator contacts the skin, enabling strong haptic cues to be delivered to the user; the lack of suitable mechanical grounding has previously been a challenge for soft haptic wearable devices.^[^ ^67^ ^]^ The device stands on the skin via two conformal spacers and can be attached with skin‐safe adhesive and/or a strap. Depending on the application and the mounting location, the spacers can be adjusted to specify the distance between the actuator's base and the user's skin. As shown in Figure 3g, a smaller distance generally results in earlier contact and larger output forces.

The 600‐µm‐thick elastomeric membrane retains the actuator stack in a non‐contact resting position and provides an additional layer of electrical insulation between the user and the device's internal components. The elastomeric layer also plays a key role in enabling high‐frequency actuation. The relaxation time of HASEL actuators is generally limited by the time the liquid dielectric takes to flow back between the electrodes;^[^ ^61^ ^]^ this time can be reduced by introducing restoring forces, therefore enabling higher actuation bandwidth. The system's quasi‐static actuation performance can be customized by changing the composition of the elastomeric membrane (Figure 4c). Softer membranes provided increased stroke, while stiffer membranes provided increased restoring force and greater durability. Since we particularly value high‐bandwidth actuation and long device lifetime, we chose the stiffest tested membrane material, which allows a free displacement of 2.44 mm at 6 kV.

Our design architecture includes multiple mechanisms that ensure the user's electrical safety while still allowing high actuation performance (Figure 4d). The actuator electrodes are fully insulated, the folding pattern of the stack provides an electrically grounded exterior, and the case and elastomeric layer encapsulate all electrical components. High‐frequency actuation requires higher current, which introduces a trade‐off between safe current limits and fast actuation speeds. Although the user is never in direct contact with the electrical components, we limit the maximum current of the power supply to 2 mA for device characterization experiments (Section 2.4) and 1 mA for all haptic cues used in the perceptual study (Section 2.5) to follow safety guidelines for magnitudes and exposure times of current passing through the body.^[^ ^68^ ^]^ In addition to the operating current, we considered the energy stored in the device, since accidental, rapid discharge of large high‐voltage capacitors can result in potentially dangerous current spikes. We calculated the maximum energy stored by our actuator stack as 16.6 mJ; high‐voltage capacitors that store less than 250 mJ of energy are not considered a hazard.^[^ ^69^ ^]^ Finally, we also employ a custom electrical circuit that shuts off the high‐voltage supply in less than 3 ms if dielectric breakdown of the actuator is detected, as described in the Supplementary Materials and Methods, as well as Table S1 and Figures S5 and S6 (Supporting Information). This circuit ensures short current exposure durations, as IEC 60479‐1 states that currents of 2 mA for a duration of up to 10 s usually produce no harmful effects.^[^ ^68^ ^]^ Beneficially, it also reduces the risk of the thermoplastic film heating up or burning due to a sustained electrical short. We further discuss electrical safety in Section 3 and in the Supporting Information.

### 2.4 Characterization of the Cutaneous Electrohydraulic Wearable Device

**Figure 5:**
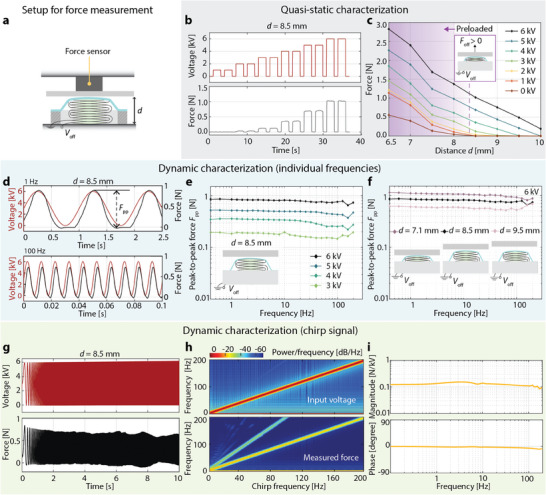
Quasi‐static and dynamic characterization of the CUTE device. a) A rigid force sensor measured normal forces at a distance *d* above the base of the membrane‐covered stack. b) Input voltage and output force for *d* = 8.5 mm. c) Mean of measured actuation forces as a function of distance and voltage. d) Input voltage and output force for 1 and 100 Hz actuation. e) Peak‐to‐peak output force for sinusoidal input signals at 0.4–200 Hz in octaves, for a distance of 8.5 mm and peak voltages from 3 to 6 kV. **f)** Peak‐to‐peak output force for sinusoidal input signals with a peak voltage of 6 kV and distances from 7.1 mm to 9.5 mm. g) Voltage signal (0–6 kV) and output force for a linear chirp test (0–200 Hz) at 8.5 mm. h) Spectrograms of the input and output in the chirp test. i) Magnitude and phase of the transfer function estimated from the chirp test.

Using a force sensor rigidly attached to a plate, we measured the output force of the device at different distances from the base when operated at voltages from 1 to 6 kV (**Figure** **5**a,b). Figure 5c shows the average measured forces for distances *d* from 6.5 to 10 mm. At *d* = 6.5 mm, the device exerts a force greater than 2.8 N under a pre‐load of 0.5 N when actuated at 6 kV; thus, it generates forces over 2.3 N. At distances larger than 8.0 mm, the device was not in contact with the plate during the off state and generated forces up to 1 N. These results reveal higher quasi‐static force output of the device at the same distance *d* compared to the results of the ten‐pouch actuator stack (Figure 3g). This difference stems from the restoring force provided by the elastomeric membrane, which compresses the relaxed ten‐pouch actuator. As a result, the device can be placed closer to the skin without making contact, which enables the actuator to operate in the regime where it can generate higher forces (i.e., at a smaller distance from the base). When the device is placed close to contact, it can generate forces higher than 1 N, and when located 2 mm away from the contact location (*d* = 10 mm), it can still generate forces of at least 0.18 N. This capability of generating forces at multiple distances demonstrates the suitability of CUTE devices for contact feedback (making and breaking contact with the skin). Even though forces are lower at higher distances for the non‐contact resting position, previous work on wearable devices on the forearm demonstrated relatively good haptic cue recognition with forces of 0.15 N.^[^ ^74^ ^]^

In addition to measuring the output forces at fixed distances, we characterized the blocking force of the device (i.e., the minimum applied force under which the device produces a stroke smaller than 1 µm) using the same experimental setup presented in Section 2.2. We prescribed forces that slowly increased over time and measured the resulting actuation stroke of the device. The results show that the device has a blocking force of approximately 16 N when actuated with 6 kV. Further information on this result appears in the Supplementary Materials and Methods and Figure S7 (Supporting Information).

The cutaneous electrohydraulic device displayed high controllability and wide‐bandwidth actuation. We tested the force output of the device in several conditions from 0.4 to 200 Hz, using positive sinusoidal input voltage waveforms. Figure 5d demonstrates device actuation forces of 0.86 N peak‐to‐peak (*F*_*pp*_) at 1 Hz actuation starting from no contact, as well as 0.75 N at the much higher frequency of 100 Hz. We tested four maximum actuation voltages (Figure 5e) and three distances from the base (Figure 5f) to demonstrate customizable performance according to the magnitude of the input voltage and the height of the device's conformal spacers. Even when the device is mounted without initial contact, application of a sinusoidal voltage causes it to contact the plate and provide peak‐to‐peak vibrational forces that are larger than 0.5 N. These results demonstrate that the amplitude of the output force and the frequency of the signal can be independently varied, and that the device is capable of achieving high actuation forces at a wide range of frequencies. Even during high‐frequency actuation at 200 Hz, we measured *F*_*pp*_ of 0.87 N when pre‐loaded (*d* = 7.1 mm), and 0.73 N when starting from a no‐contact position (*d* = 8.5 mm), which represents less than 14% force reduction compared to its output at 1 Hz.

To test the frequency content of the response, we measured the output force while driving the actuator with a linear chirp from 0 to 200 Hz over ten seconds. Figure 5g shows the measured input voltage and force output, while Figure 5h shows the power spectral density of the input voltage and the output force; the definition of power in dB used in this analysis is described in the Supporting Information. The results indicate that the device provides a clean response at the desired frequency throughout the tested range, with no resonances at lower frequencies and a minimal response at double the desired frequency (14 dB smaller). The device can also actuate at frequencies above 200 Hz; we tested it up to 500 Hz (Figure S8, Supporting Information), measuring substantial force output, though the measured peak voltage partially deviated from the prescribed peak voltage at frequencies above 200 Hz, especially for higher peak‐to‐peak voltages. Further, we perform a transfer function estimate of the output force over the input voltage from 0 to 200 Hz (Figure 5i), which shows that the device has a relatively flat output across frequencies and corroborates that the system does not have strong resonances or antiresonances. This clean and controllable response makes the actuator suitable for delivering crisp haptic sensations at precise frequencies.

A key benefit of electrohydraulic actuators is the low power they require to hold their actuation state;^[^ ^59^, ^60^ ^]^ our device draws only 3.0 mW when holding an extended position (Figure S9, Supporting Information). This low power consumption leads to minimal heating of the device. To demonstrate this safety and comfort advantage, we measured the temperature of the device during sustained low‐frequency actuation and observed no temperature increase (Movie S2, Supporting Information). In contrast, Movie S2 (Supporting Information) shows that a similarly sized voice‐coil actuator commonly used in haptics undergoes rapid heating when driven with a zero‐mean sinusoidal voltage at the same frequency.

### 2.5 Perceptual Study Evaluating the Cutaneous Electrohydraulic Wearable Device

We conducted a perceptual study to evaluate the CUTE device's ability to deliver haptic cues to the hairy skin on the dorsal side of a user's non‐dominant wrist, which is a typical location for long‐term wearable devices (e.g., wristwatches). The perceptual study consisted of two tasks plus an evaluation of the device. The cue identification task aimed to determine how well humans can recognize a diverse set of haptic cues provided by the device. The cue description task sought to evaluate the qualitative perception of a broader set of the device's haptic cues.

**Figure 6:**
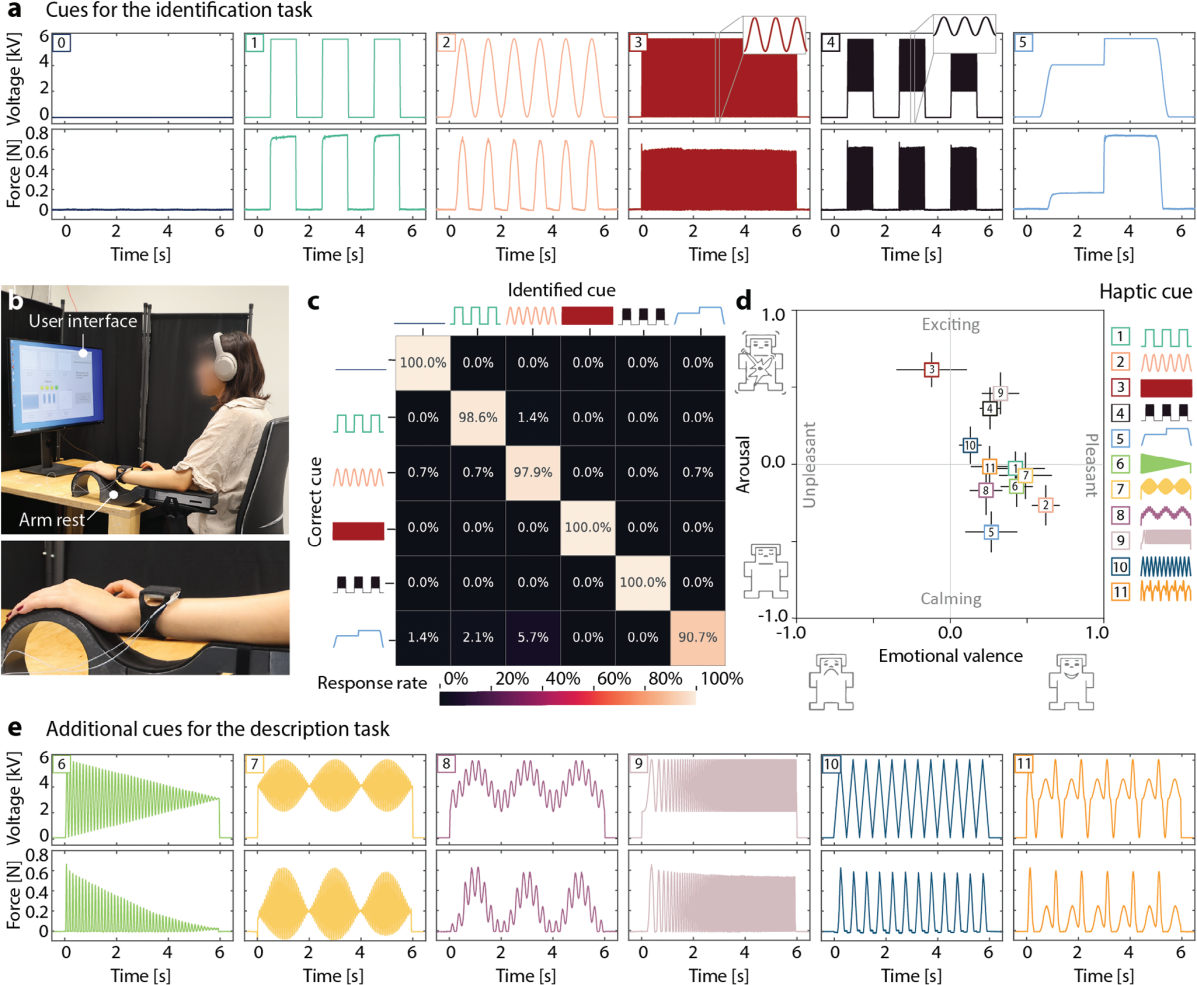
Design and results of the perceptual study revealing that CUTE haptic sensations are easily recognized and are almost all perceived as pleasant. a) Voltage waveform (top) and corresponding measured force (bottom) for the six cues used in the cue identification task; cue 0 is a reference cue of constant zero voltage (no actuation). b) User interface and experimental setup. c) Results of the cue identification task: three cues were identified perfectly, and the other three were recognized more than 90% of the time, for an overall recognition accuracy of 97.9%. The identified cue significantly depends on the delivered cue, χ^2^(25, n = 840) = 3992.64, p < 0.0001. d) Results of the cue description task for the 11 active cues tested by the participants; the perpendicular line segments show the means and standard errors (n = 14) for the perceived emotional valence and arousal of each cue. Only the sustained vibration (cue 3) is rated unpleasant. e) Voltage waveform (top) and corresponding measured force (bottom) for the six additional cues used in the cue description task.

**Figure** **6**b shows the experimental setup for the perceptual study, which included an armrest for the user's non‐dominant arm, a wireless mouse, a screen, and noise‐canceling headphones playing pink noise (to mask environmental noise). We powered the device from a current‐limited high‐voltage amplifier with custom safety hardware for automatic shut‐off as described in Section 2.3. We recruited fourteen participants (7 females and 7 males) ranging in age from 22 to 52 years old (mean age of 31 ± 7). The participants had widely varying levels of experience with haptic devices, and half of them had previously worn a commercial haptic‐feedback‐enabled device on their wrist.

#### 2.5.1 Cue Identification Task

For each trial of the cue identification task, a haptic cue was delivered by the device, and the participant was asked to identify it by selecting the visual depiction of the corresponding voltage waveform. Six haptic cues, each 6 s long, were used. The five active cues were made of different combinations of sinusoidal and square signals with frequencies ranging from 0.5 to 25 Hz and a maximum voltage of 6 kV. An additional cue of constant zero voltage was added as a reference. Figure 6a shows the voltage waveforms of these haptic cues and their generated forces when actuated at a non‐contact resting position against a rigid plate (further described in Tables S2 and S3, Figures S10–S14, and Movie S3, Supporting Information).

The cue identification task consisted of three sequential phases. In the first, participants had a free practice phase of up to three minutes to learn the haptic cues: when they clicked the visual waveform for a cue, that cue would play. Next, they practiced the cue identification task, receiving immediate visual feedback indicating which cue had been presented. Each cue was presented twice in random order. Then, in the last phase, participants were asked to identify each of the six cues ten times in random order (for a total of 60 trials) divided into four sets of 15 cues. A short break was provided after each set. The number of cues and repetitions used in this task were chosen in accordance with previous studies^[^ ^77^, ^78^ ^]^ and were designed to balance sufficient sample size with total experiment time for each participant.

Participants achieved an overall accuracy of 97.9%, with six of fourteen achieving perfect cue recognition. A chi‐square test of independence was conducted to further examine these results, χ^2^(25, N = 840) = 3992.64, p < 0.0001, indicating that the identified cue significantly depends on the delivered haptic cue, rejecting the null hypothesis. Three cues (cues 0, 3, and 4) were identified with 100% accuracy (out of 140 trials per cue), as shown in Figure 6c. The 0.5 Hz square wave (cue 1) and 1 Hz sinusoid (cue 2) had high recognition rates of 98.6%, and 97.9%, respectively. Cue 5 had the lowest recognition rate (90.7%); it was misidentified as cue 0 (1.4%), cue 1 (2.1%), or cue 2 (5.7%). These mistakes could be due to the low number of contact changes in the cue and/or its similarity with the other cues; cue 5 has sinusoidal actuation at the beginning and end, similar to cue 2, as well as a sharp step similar to cue 1.

#### 2.5.2 Cue Description Task

In the cue description task, participants experienced the five active cues of the previous task as well as six additional cues with frequencies ranging from 0.5 to 40 Hz. They rated each cue's emotional valence (from –1: “unpleasant” to 1: “pleasant”) and arousal (from –1: “calming” to 1: “exciting”) using scale bars. Figure 6d shows the mean and standard error of these ratings in a circumplex model graph.^[^ ^64^ ^]^ The six additional haptic cues for this task are shown in Figure 6e and in Figures S15–S20, Tables S3 and S4, and Movie S4 (Supporting Information). The forces measured for these haptic cues illustrate the large design space for haptic cues possible with the CUTE device, including variable frequencies and tunable contact forces.

The results of the arousal ratings show that our device provides haptic cues that evoke sensations ranging from exciting to calming. The perceived arousal seems to be directly related to the slew rate of the actuation signal, i.e., the rate of change of the applied voltage. Specifically, vibrotactile cues that had higher frequencies (above 24 Hz) were found to be exciting (cue 3: 0.61 ± 0.11; cue 4: 0.36 ± 0.14; cue 9: 0.46 ± 0.14). Cue 5, which had a low slew rate and the lowest number of distinct contact changes, was found to be the most calming (–0.44 ± 0.13). On the other hand, the valence ratings showed that all cues aside from cue 3 (–0.12 ± 0.23) were perceived as pleasant. In particular, haptic cues with slow and smooth changes were rated as more pleasant, with the highest rating given to cue 2 (0.62 ± 0.09).

To further evaluate the expressiveness of this approach to cutaneous feedback, we also asked participants to describe how each cue felt. The results of these open‐ended questions confirmed that the device is capable of providing haptic cues that evoke a variety of different sensations. For example, most participants associated the two cues with higher frequencies with alarms and alerts (10/14 = 71.43% for cue 3; 9/14 = 64.3% for cue 4). Cues with a regular pattern and a slow transition in making and breaking contact with the skin were described with terms related to aliveness, such as “heartbeat,” “lifelike”, and “animals” (42.9% for cues 2 and 10; 50% for cue 11). Cue 6, which had a decreasing amplitude, was associated with devices turning off (42.9%), while cue 9, a chirp with increasing frequency, was associated with engines (64.3%) and devices starting up (78.9%). Finally, the cues that included sustained pressure and slow motion (0.5–1 Hz) were described as gentle and soft (cue 5: 28.6%; cue 8: 35.7%), while cue 1, which had sustained pressure and rapid transitions, was associated with touch, tapping, and notifications by six participants (42.9%) and with clocks by seven participants (50%). Additional descriptions of the haptic cues given by participants are included in Tables S2–S4 (Supporting Information).

#### 2.5.3 Device Evaluation

After completing both tasks, participants rated the design of the device and its ability to deliver haptic stimuli on a scale bar from 0: “strongly disagree” to 1: “strongly agree.” Participants found the device highly comfortable to wear (0.91 ± 0.06), and they could feel the start (0.80 ± 0.07) and end (0.72 ± 0.06) of contact between the actuator and their skin as well as how strongly the actuator was pushing (0.78 ± 0.08).

In the final open‐ended survey question, participants commented positively on the wide range of haptic sensations that the device can evoke. Almost half of the participants who had previous experience with wrist‐worn devices (3/7 = 42.8%) commented that the device was lighter and at least as comfortable as commercially available wrist‐worn devices. Four participants (28.6%) commented that the perception of the cue was affected by the speed at which the contact with the skin was made. Thus, the slow transitions of the sinusoidal waveforms (e.g., cues 2 and 5) were more difficult to feel than sharp changes of similar magnitude (e.g., cue 1).

## 3 Discussion and Conclusion

Here, we introduced a system architecture for a new class of compact haptic devices designed to deliver salient, distinguishable, and pleasant cutaneous sensations to the hairy skin. To achieve this performance, we leverage a custom materials design for electrohydraulic actuators that can safely contact the skin and have a small footprint; this materials design unlocks a unique set of performance attributes essential for robust haptic feedback – namely, controllable actuation from constant output to high frequencies, variable amplitude, clean frequency response, high output force, and large stroke. Our mounting strategy provides a method to harness these performance attributes to make and break contact with the skin, transfer forces effectively, and apply more complex feedback combining multiple modes of mechanical stimuli. To highlight the advantages of our approach, **Table** 1 compares CUTE devices to other technologies previously used for haptic actuation on hairy skin. Only cutaneous electrohydraulic actuation can provide highly expressive and pleasant haptic sensations; importantly, our approach also delivers high user safety and facilitates system integration.

**Table 1:** Table comparing key attributes of haptic actuation technologies for use on the hairy skin.

		***Expressiveness of haptic sensations***							***Safety***		***System integration***				
	**Actuator**	Sufficient stroke for contact feedback	Capability to sustain high normal forces on skin	Low‐frequency actuation (<10 Hz)	Mid‐frequency vibration (10–80 Hz)	High‐frequency vibration (>80 Hz)	Independent control of amplitude and frequency	Capability to deliver pleasant sensations	Electrical safety (low current)	Thermal safety (staying cool)	Compactness of devices for hairy skin	Portability of driving mechanism	Quiet operation	Acquirability	Sample wearable haptic devices for hairy skin
**Electromagnetic**	**ERM motor**	No	No	No	Medium	High	No	Low	Medium	Medium	High	Very high	Medium	Very high	^[^ ^70^, ^71^ ^]^
	**LRA**	No	No	No	Low	High	Low	Low	Medium	Medium	High	Very high	Medium	Very high	^[^ ^72^, ^73^ ^]^
	**Voice coil**	Medium	High	High	High	High	High	Medium	Low	No	Low	Medium	Medium	High	^[^ ^30^, ^74^ ^]^
	**Servo motor**	High	High	High	Low	No	High	Low	Medium	Medium	Low	Medium	Low	Very high	^[^ ^25^, ^75^ ^]^
**Fluidic**	**Pneumatic actuator**	High	Very high	High	Low	No	High	High	Very high	High	High	Low	Low	Medium	^[^ ^35^, ^38^ ^]^
**Electrostatic**	**Piezoelectric actuator**	No	Low	Medium	High	High	High	Low	High	High	High	High	High	High	^[^ ^76^ ^]^
	**DEA**	Low	Medium	High	High	Medium	High	Medium	Medium	Medium	High	High	High	Medium	^[^ ^50^ ^]^
**Electrohydraulic**	**HAXEL**	Low	Medium	High	Medium	Medium	High	Medium	High	High	Very high	High	High	Low	^[^ ^63^ ^]^
	**CUTE**	High	High	High	High	High	High	Very high	High	High	High	High	High	Medium	**This work**

The electrohydraulic actuation unit we employed can be tailored to the desired performance. Higher displacement can be achieved by stacking more pouches, while force can be increased by using wider electrodes and/or materials systems with higher permittivity.^[^ ^57^, ^79^ ^]^ Increasing permittivity would allow actuators with similar performance to operate at a lower voltage at the expense of higher current requirements. Output forces and displacements can also be tuned using electrohydraulic principles. For example, filling each pouch with less liquid dielectric would produce higher forces but lower displacements, as shown in Figure S3 (Supporting Information). Alternatively, stacks of fewer pouches or single‐pouch actuators would provide less displacement while maintaining high‐force and broad‐bandwidth actuation. Such a design could be suitable for delivering localized and strong haptic cues to glabrous skin, which has higher sensitivity. Due to the typical force‐displacement behavior of HASEL actuators, there is a trade‐off between device designs that provide contact feedback (requiring a non‐contact resting position away from the skin) and designs that generate larger forces, since higher forces are typically generated at lower displacements. For applications where only vibration or sustained forces are desired, placing the device closer to or in constant contact with the skin, or employing additional stacked pouches, could enable larger output forces.

Since electrohydraulic actuators are electrically driven and use high voltages and low currents for actuation, their driving mechanism carries both benefits and drawbacks. They can be actuated by a pocket‐sized, battery‐powered high‐voltage amplifier,^[^ ^44^ ^]^ which draws a strong contrast to the pumps, valves, and tubes required by most soft pneumatic haptic devices. The use of high voltage for powering actuators often raises safety concerns, as the potential risks of low‐power, high‐voltage systems are not widely understood by potential users. In fact, it is the amount of current and the length of time the current passes through the body that can cause harm and introduce the risk of ventricular fibrillation (especially at frequencies from 50 to 60 Hz).^[^ ^68^, ^69^, ^80^, ^81^ ^]^ We have therefore limited the maximum current of the device, and future compact high‐voltage electronics designed for use in contact with the human body should also enforce safe limits on current magnitude and exposure time. Electrohydraulic zipping actuators behave like variable capacitors and therefore store electrical energy. While it is possible for electrostatic systems to deliver dangerously high currents through rapid discharge of electrical energy stored in high‐voltage capacitors, the safe limit for energy stored^[^ ^69^ ^]^ is fifteen times higher than the energy stored in the CUTE actuators. Future systems using very large actuators or connecting multiple units together should include peak current limits, fuses, or other techniques to prevent simultaneous discharge of large capacitive loads.^[^ ^55^ ^]^

We instantiated our system architecture through a prototypical CUTE device that delivers richly varying tactile sensations to the hairy skin in a compact, lightweight, and wearable form factor. The near‐perfect cue recognition rates of the perceptual study suggest that this device effectively transforms various actuation waveforms into diverse and recognizable cutaneous sensations that can easily be identified even on the wrist, which has low tactile sensitivity.^[^ ^82^ ^]^ These results indicate that such devices would be suitable for delivering distinguishable haptic cues on other parts of the body with similar sensitivity, such as the upper arm or the lower back.^[^ ^83^ ^]^

The cue ratings indicate that participants experienced almost uniformly pleasant broad‐bandwidth cutaneous sensations during the study. The material composition of the device plays an important role in its pleasant perception: the actuator, elastomeric membrane, and conformal spacer feel soft to the touch due to their mechanical compliance, and the actuator's low thermal conductivity, low thermal capacitance, and low heat generation minimize heat transfer with the skin. The pleasantness of the cues also stems from our system's ability to produce tactile sensations that have rarely been output by wearable devices (contact feedback, slowly changing pressure), resulting in evoked sensations that feel far more natural than pure vibrotactile stimuli. Such sensations could also stimulate C‐tactile afferents that are associated with the perception of pleasant touch and typically respond to slow, low‐force mechanical stimuli.^[^ ^84^ ^]^ Additionally, the contact feedback enabled by the device may be a key contributing factor to the high accuracy rate observed in the cue identification task. Specifically, the dynamic changes in tactile stimuli, such as the start and end of contact, trigger both the fast‐ and slow‐adapting types of Aβ fibers in the hairy skin.^[^ ^85^ ^]^ Consequently, contact feedback should result in a stronger neural response compared to static touch and should limit sensory adaptation, which typically increases the threshold required for perceiving successive haptic stimuli.^[^ ^86^ ^]^

Furthermore, the haptic cues from our study mainly occupy the first (exciting/pleasant) and fourth (calming/pleasant) quadrants of the valence‐arousal circumplex model. The combined space of these two quadrants is seldom reached by previous work using only vibrotactile feedback.^[^ ^40^, ^87^, ^88^ ^]^ These results demonstrate the value of our system architecture as a method to produce compelling haptic cues for diverse applications, such as non‐urgent notifications and haptic guidance.

In addition, the high controllability of the system and the customizability of its haptic cues enable unprecedented design freedom to explore new cutaneous sensations, which can be used to advance the study of human haptic perception. Since the capacitance of an electrohydraulic zipping actuator depends on its deformation and changes repeatably during actuation, these devices could simultaneously function as actuators and capacitive sensors.^[^ ^53^, ^89^, ^90^ ^]^ This principle of self‐sensing could be implemented in our cutaneous devices to provide more precise contact timing and more advanced haptic sensations through closed‐loop control. Furthermore, the high degree of control over the actuator output could be used to provide haptic sensations that can be adjusted according to user sensitivity, which varies across frequencies and body locations. Immersive feedback across the body could be created by more advanced garments containing arrays of CUTE actuators.

In summary, this class of haptic devices provides a unique way to evoke a wide range of salient sensations, from lifelike and emotive touch to exciting and powerful vibrations. This unprecedented haptic actuation capability can enable richer immersion in AR/VR, complementing visual and audio cues in new ways, and holds great potential for discreetly sending information to the user through their skin.

## 4 Experimental Section

### Materials

The electrohydraulic actuators were fabricated from a BoPET film with a single‐sided heat‐sealing layer (Mylar Petroplast 850H, DuPont), the electrodes were screen‐printed with silver ink (ECI‐1011, Henkel), and an adhesive PET film was applied for the insulation later (Polyester Film Electrical Tape 5, 3M). Each pouch was filled with silicone oil (Silicone oil M5, Carl Roth). Copper tape (6.35 mm Oubaka copper tape, Oubaka) was applied to the leads of the electrodes and secured with a drop of conductive carbon glue (16056 DAG‐T‐502 Carbon Paint, Ted Pella) to ensure a robust electrical connection.

The body of the device was fabricated using a multi‐material 3D printer (J850, Stratasys) using rigid material (Vero PureWhite, Stratasys) for the housing and conformal material (Agilus30 and VeroUltraClear, Stratasys) for the spacers. The elastomeric membrane was cast from three types of two‐part silicone elastomer (E10: Ecoflex 00‐10, E30: Ecoflex 00‐30, and E50: Ecoflex 00‐50, Smooth‐On) using a bladecaster (Zehntner ZAA2300 & ZUA2000, Proceq) set to a thickness of 600 µm. Insulated high‐voltage cables (HFP‐1828‐19‐10, hivolt) were added and disposable skin‐safe adhesive film (2477P, 3M) was used to adhere the device spacers to the skin. The device strap was constructed using soft loop fabric (Polyamide Velour, Extremtextil), stretchable fabric (stretch bengaline, fabfab), and hook‐tape (molded Velcro hook‐tape, Extremtextil).

### Methods

For all experiments, the actuator was driven with a high‐voltage amplifier (TREK 610E, TREK 10/10B‐HS, or TREK 50/12, Advanced Energy) controlled by Matlab (R2022a, The Mathworks) via a data acquisition (DAQ) device (USB 6212‐BNC, National Instruments).

For the quasi‐static force‐displacement experiments (Figure 3), the force of the actuator was measured using a displacement‐controlled dual‐mode muscle lever (310C‐LR, Aurora Scientific). For static and dynamic characterization of the device (Figure 5), the forces were measured using a calibrated force sensor (Nano17, ATI Industrial Automation). Power measurements were conducted by measuring the voltage using the built‐in voltage monitor of the high‐voltage amplifier (TREK 50/12, Advanced Energy) and simultaneously measuring the current on the ground side of the actuator using an electrometer (Model 6514, Keithley). Displacement measurements were done with a laser displacement sensor (LK‐H157, Keyence). The movies in the Supporting Information were recorded using a camera (Canon EOS R5), a voice‐coil actuator (Haptuator Redesign, Tactile Labs), an infrared camera (VarioCAM HD, InfraTec), and a high‐speed camera (Phantom v2640, Vision Research).

### Perceptual Study

The perceptual study employed the high‐voltage amplifier TREK 50/12 (Advanced Energy) and a safety circuit further described in the Supplementary Materials and Methods (Supporting Information). This human‐subject experiment was approved by the Max Planck Society's Ethics Council under the framework agreement of the Haptic Intelligence Department (protocol number F030A). All participants provided written informed consent; those who were not employed by the Max Planck Society were compensated at minimum wage for the duration of the study (on average 15 euros for 80 min of participation time).

### Statistical Analysis

For the data presented in Figure 3, we computed the mean and standard deviation (SD) using Matlab for both the single‐pouch and ten‐pouch actuators. For each displacement, three different actuators were tested for three cycles each, yielding a total sample size of n = 9.

A chi‐squared test of independence was performed on the results of the cue identification task using Python to determine if there was any dependency between the delivered and perceived cues. The χ^2^ and p values were reported for a sample size of n = 840, degrees of freedom = 25. Statistical significance was set at a family‐wise error rate of α = 0.05.

The cue description results presented in Figure 6d report the mean and standard error (SE) of the valence and arousal scores given by the participants (n = 14) for each haptic cue.

## Conflict of Interest

C.K. is listed as an inventor on three patents that cover fundamentals and basic designs of HASEL actuators (Assignee of all three patents is “The Regents of the University of Colorado”: US Patent 10995779B2, granted May 4, 2021; US Patent 11486421B2, granted November 1, 2022; US Patent 11408452B2, granted August 9, 2022). C.K. is a cofounder of Artimus Robotics, a start‐up company that commercializes HASEL actuators. The authors declare no other competing interests.

## Author Contributions

N.S., C.K, and K.J.K. conceived the research. N.S. and Z.Y. designed and fabricated the device, designed experimental setups, and characterized the actuators and device. P.R. provided guidance on electrohydraulic actuator design and characterization methods. N.S., G.B., and K.J.K. designed the perceptual study. N.S. and G.B. conducted the perceptual study. Z.Y. developed the safety electronics for automatic shut‐off and the Matlab interface for the perceptual study. N.S., G.B., Z.Y., and P.R. analyzed the results. C.K. and K.J.K. supervised the research. N.S. and Z.Y. drafted the manuscript and figures, and all authors revised them.

## Supporting information

Supporting Information

Supplemental Movie 1

Supplemental Movie 2

Supplemental Movie 3

Supplemental Movie 4

## Data Availability

The data that support the findings of this study are openly available in Edmond at https://doi.org/10.17617/3.E6GUSA.
