# Supplementary material for: Cutaneous Electrohydraulic (CUTE) Wearable Devices for Pleasant Broad‐Bandwidth Haptic Cues
Source: Adv Sci (Weinh). 2024 Sep 6;11(48):2402461. doi: 10.1002/advs.202402461 (PMC11672320; doi:10.1002/advs.202402461)
Supplement: Supplementary file 1 — Supporting Information [file ADVS-11-2402461-s003.pdf]

ADVANCED SCIENCE
Open Access

Supporting Information

for *Adv. Sci.*, DOI 10.1002/advs.202402461

Cutaneous Electrohydraulic (CUTE) Wearable Devices for Pleasant Broad-Bandwidth Haptic Cues

Natalia Sanchez-Tamayo, Zachary Yoder, Philipp Rothemund, Giulia Ballardini, Christoph Keplinger and Katherine J. Kuchenbecker**

Supporting Information

Cutaneous Electrohydraulic (CUTE) Wearable Devices for Pleasant Broad-Bandwidth Haptic Cues

Natalia Sanchez-Tamayo, Zachary Yoder, Philipp Rothemund, Giulia Ballardini, Christoph Keplinger, and Katherine J. Kuchenbecker**

** Co-corresponding authors.* E-mail: ck@is.mpg.de, kjk@is.mpg.de

This document includes:

Supplementary Materials and Methods
Figures S1 to S20
Tables S1 to S4
Captions for Supplementary Movies S1-S4

Other supplementary materials for this manuscript include:

Movies S1 to S4

The data and code used in the analysis of this work can be found at the following link:
https://doi.org/10.17617/3.E6GUSA

Supplementary Materials and Methods

Fabrication of Electrohydraulic Actuators

The electrohydraulic actuators were fabricated using the process depicted in Figure S2, which is a modified version of the process proposed by Mitchell et al. [1] for HASEL fabrication. We first fabricated a strip of 10 unfilled soft electrohydraulic zipping pouches by heat-sealing two sheets of 15-μm-thick BoPET (Mylar 850H, Petro Plast) using a modified CNC machine (Carbide 3D Shapeoko XL, Shapeoko) with a 1-mm-diameter heat-sealing tip set to 260°C, as shown in Figure S2a. Electrodes are screen-printed (Figure S2b) on each side of the strip of pouches using silver conductive ink (ECI-1011, Henkel) and cured in an oven at 65°C for 12 minutes.

We employed silver conductive ink because it has a sheet resistance ($< 0.005\,\Omega$/sq for 25 μm thickness) that is 10 000 times lower than the carbon screen-printable ink ($< 50\,\Omega$/sq for 25 μm thickness) previously used for HASEL actuators [2, 3]. Low-resistance electrodes are an important, innovative solution to prevent heating of the thin electrical connections in actuators with multiple units connected in series and actuated at high frequency, such as in small haptic devices. Each 14 mm × 14 mm pouch has two rectangular 13.5 mm × 3.5 mm electrodes per side; we reduced the length of each electrode to be 0.5 mm less than the pouch width in the direction of the lead connections to prevent the electrode from overlapping the heat-sealed regions, which could increase the likelihood of electrical breakdown. We also observed that actuating the device with reverse polarity resulted in faster actuator electrical breakdown than employing singular polarity.

We laser cut 60-μm-thick insulating PET adhesive films (Polyester Film Electrical Tape 5, 3M). One layer of PET film was placed on each side of the strip, covering all electrode areas but avoiding the electrode-free areas (Figure S2c). Then, the 10-pouch actuator strip was filled with 0.8 ml of liquid dielectric (5 cSt silicone oil, Roth), which we distributed between the pouches by manually tensioning the film while it was lying flat. Next, we heat-sealed the connections between the pouches with a soldering iron to prevent flow between neighboring pouches, and we laser cut the excess film (Figure S2d) around and between the pouches to reduce the mechanical constraints on actuation caused by excess material, which can reduce actuator performance [4]. We then stacked the actuators with transfer tape (950, 3M) and connected the actuator leads with copper tape (6.35 mm Oubaka copper tape, Oubaka) using carbon ink (16056 DAG-T-502 Carbon Paint, Ted Pella) to create a robust electrical connection.

Fabrication of the Device

The case of the device was fabricated using a multi-material 3D printer (J850, Stratasys): a hard material was used for the housing and internal bracket (Vero PureWhite, Stratasys), and a softer material was used for the conformal spacer (mixture of Agilus30 and VeroUltraClear, Stratasys). The base of the housing was designed with a breathing channel to allow free air exchange between the inside and outside of the device, facilitating the expansion of the actuator; closure of this channel greatly inhibits device actuation. The device is connected to the voltage supply by two 1 mm diameter insulated cables rated for >18 kV (HFP-1828-19-10, hivolt), which are soldered to two fixed copper pads at the base of the housing.

To assemble the device as shown in Figure 4a, we place the actuator inside the 3D-printed rigid housing and secure the pairing of the copper pads between the actuator and the housing with PET adhesive tape (Polyester Film Electrical Tape 5, 3M). This pairing is not permanent and facilitates the replacement of the actuator. We then place the insulating elastomeric membrane (Ecoflex 00-10, 00-30, or 00-50, Smooth-On) on top of the internal bracket, and we clamp it between the stiff internal bracket and the rigid housing without any pre-stretch. The elastomeric membranes are fabricated using a blade caster (Zehntner ZAA2300 & ZUA2000, Proceq) set to 600 μm thickness and 1.3 mm/s speed.

To fabricate the strap, we laser cut the strap pattern from two materials: soft stretchable fabric (stretch bengaline, fabfab) and soft loop fabric (Polyamide Velour, Extremtextil). We sew the edges of both laser-cut parts together and create two pockets for placing the 3D-printed clamps, which retain the fabric strap to the device. We attach a thin strip of hook-tape (molded Velcro hook-tape, Extremtextil) to the fabric strap for fastening the strap around the user's wrist. We press-fit M2 nylon nuts (RS PRO) inside the 3D-printed clamps and slide the clamps into the aforementioned pockets. Finally, we close the device by threading four M2 nylon screws (RS PRO) through the case and into the M2 nuts in the clamps.

Actuator and Device Characterization

Characterization of Actuator Performance

To characterize the force of the actuators throughout their displacement range, we employed a dual-mode muscle lever (310-LR, Aurora Scientific) to prescribe a displacement profile consisting of 40 logarithmically spaced positions while simultaneously measuring forces and displacements. A maximum distance of 1.8 mm was used for a single pouch and 17.6 mm for a stack of 10 pouches. We adapted the dual-mode muscle lever to create linear motion through a connecting rod and a slider mechanism, as shown in Figure S4a. At each prescribed distance, the force was measured while the selected actuator was repeatedly turned on and off with a cycle duration of 4 seconds (2 seconds on). The actuator was driven with a high-voltage amplifier (TREK 50/12, Advanced Energy) using ramped-edge square waves with amplitudes of 3 kV, 4 kV, 5 kV, or 6 kV and ramp times of 50 ms. The force was measured at each voltage level, and the weights of the connecting rod and slider were subtracted from all measurements. The dual-mode muscle lever was force-limited to 20 N for all tests (i.e., if the force needed to reach a given displacement exceeded 20 N, the muscle tester actively changed the displacement to keep the force at a maximum of 20 N).

To compute the force-distance curves, we eliminated the first 0.5 s and last 0.2 s of the recorded force data for each voltage-on and voltage-off step to eliminate the transient response of the actuator. Then, we took the average of the remaining 1.3 s of force data and computed the difference between the voltage-on and voltage-off values to find the generated force. For each displacement step and each actuator tested, we cycled the voltage on and off five times and eliminated the first and last cycles because the dual-mode muscle lever was changing its displacement at the very beginning or end of those steps. This procedure left three cycles to be analyzed at each displacement value for each actuator tested.

Measurement of Device Displacement

The displacement of the device equipped with different elastomeric membranes was measured using a laser displacement sensor (LK-H157, Keyence) for square voltage signals (2 seconds on, 2 seconds off) at 6 kV, 5 kV, 4 kV, 3 kV, 2 kV, and 1 kV, measured one time each. The actuator was driven with a high-voltage amplifier (TREK 610E, Advanced Energy). The displacement data was filtered with a median filter, and the average of the measured displacement was reported at each voltage activation minus the displacement at 0 kV.

Electrical Safety of the Device

We elucidate several considerations for the safe operation of high-voltage wearable devices and design four layers of safety into our system to ensure compliance with relevant engineering standards.

First, the PET insulating films and device housing alone provide sufficient electrical insulation to make the device safe to wear. The 60-μm-thick PET adhesive film used for electrical insulation is four times thicker than the 15-μm-thick BoPET pouch film. Thus, the electrical breakdown voltage to the outside of the pouch is several times higher than the electrical breakdown voltage through the actuator film.

This design ensures that the eventual electrical breakdown of the actuator occurs through the actuator film, safely confining the electrical failure inside the encapsulation.

Second, we analyzed the maximum stored energy of the actuator. Since HASEL actuators operate as variable capacitors at high voltage, they store electrical energy. Should a person come into direct contact with such an energized capacitor, the stored energy could discharge rapidly through the person resulting in potentially dangerous electrical currents when the stored energy is large. NFPA 70E dictates that high-voltage capacitors (over 400 V) are not considered a hazard if they store less than 250 mJ of electrical energy [5]. The energy stored by a parallel-plate capacitor can be described by $\frac{1}{2}CV^2$. Here, the applied voltage is V, and the capacitance is $C = \varepsilon_r \varepsilon_0 \frac{A}{d}$, where ε_r is the permittivity of the film, ε_0 is the vacuum permittivity, A is the electrode area, and d is the distance between the electrodes, equal to twice the thickness of the film. With a permittivity $\varepsilon_r = 3.3$, our actuator therefore has a maximum capacitance (when the electrodes are fully zipped) of 0.92 nF. With a maximum operating voltage of 6 kV, the maximum energy stored in our system is 16.6 mJ, which is still fifteen times lower than the safe limit.

Third, we considered the maximum current that can pass through the human body without harmful consequences, as described in IEC 60479-1. This standard considers both the magnitude of the current and the duration of exposure to the human body and explains potential adverse effects. It considers both direct current (DC) and alternating current (AC) in the 15–100 Hz range: we base our analysis on the alternating current case because the limits for AC are more conservative. The standard states that currents under 5 mA usually produce “no harmful electrical physiological effects” even with long exposure durations (10 s) [6]. It states the same for much higher current magnitudes with shorter exposure times (up to 200 mA for 10 ms, for example). Our high-voltage amplifiers (TREK 50/12 or Trek 610E, Advanced Energy) have built-in current limiting functionality, which makes them a convenient tool for following this standard. We limited the maximum current to 1 mA during the perceptual study and to 2 mA for characterization experiments. Limiting the operating current to below the safe limit may affect performance during high-voltage, high-frequency actuation (above 200 Hz for this actuator design), as faster actuation requires faster charging and thus higher currents.

Fourth, we introduced a safety circuit (Figure S5) that can shut down the high-voltage output in just 3 ms and discharge the device in any circumstances that cause the high-voltage output to differ from the expected output. The purpose of this additional safety circuit was to detect electrical breakdown of the electrohydraulic actuator, which creates a short-circuit from high voltage to ground. Swiftly turning off the voltage output both prevents further damage and heating of the film during breakdown and serves as an indicator that the actuator needs to be replaced. The circuit also shuts off the voltage during cases of electrical arcing to the atmosphere, unexpected rapid charging or discharging, and when the high-voltage amplifier is not able to charge and discharge the device quickly enough to meet the prescribed voltage at high actuation frequencies.

This functionality leverages the “out-of-regulation status” indicator of the TREK high-voltage amplifier, which indicates when the output voltage differs from the prescribed voltage. This signal serves as an input to our circuit; when the out-of-regulation status is indicated (logic LOW), the circuit disables the high-voltage output of the TREK and closes a high-voltage relay, which shorts the actuator to electrical ground, thus discharging the actuator and providing a direct, low-resistance electrical path to ground. The circuit then holds its state until a manual reset button is pressed. We tested the reaction speed of the safety circuit (Figure S6) and found that it fully discharged the device in less than 3 ms. Future versions of the circuit could replace R2 and R3 with a 5 V low-dropout (LDO) regulator to generate the 5 V powering the two integrated circuits.

Characterization of Device Performance

For the device characterization experiments, the actuator was driven by a TREK 610E (Advanced Energy) high-voltage amplifier, and forces were measured using a six-axis force-torque sensor (Nano17, ATI

Industrial Automation) attached to a rigid and height-adjustable plate. The baseline of the force sensor was set to zero when there was no contact, so the force measured at 0 kV was the pre-load on the device.

Quasi-static Characterization of the Device

The static force generated by the device was measured at multiple distances from the base and filtered using a median filter. The quasi-static forces were calculated as the average during the actuation of two cycles at 1 kV, 2 kV, 3 kV, 4 kV, 5 kV, or 6 kV.

Dynamic Characterization of the Device

To calculate the peak-to-peak force, we measured five seconds of continuous sinusoidal actuation at each frequency and discarded the first second of actuation to eliminate start-up transients. The normal force was filtered with a median filter, and the peak-to-peak forces were calculated as the maximum force minus the minimum force measured. For frequencies above 99 Hz, the peak-to-peak forces were calculated over ten actuation cycles, and for lower actuation frequencies, peak-to-peak forces were calculated over 3.5 seconds of actuation.

To measure the frequency response, we ran a ten-second-long linear chirp signal from 0 Hz to 200 Hz for three consecutive repetitions and used the second repetition for the dynamic analysis. The power spectral density of the input voltage and output force were plotted using the *spectrogram* function in Matlab R2022a (The Mathworks). The spectrogram is plotted in dB/Hz using the decibel definition $\text{dB} = 10\log_{10} P/P_0$, where the power reference P_0 is 1 kV for the voltage and 1 N for the output force.

Since the Maxwell stress that drives HASEL actuators is proportional to the square of the applied electric field, using a zero-mean sinusoidal voltage signal would cause the output frequency of the device to double. Thus, we employ a positive polarity signal from 0–6 kV to actuate the device. In this case, the input signal is not centered around 0 kV. Similarly, the output force of the device is not centered around 0 N but rather has a non-constant bias that changes with the driving frequency. Thus, we de-trended both the input voltage and the output force of the device to analyze only the oscillating component and remove the effects of both the voltage bias and the resulting force bias. Using the same data, we calculate the transfer function estimate using the *tfestimate* Matlab function, which uses Welch's average periodogram method.

Force-Stroke Curves of the Device

The force-stroke curves of the device were measured with a dual-mode muscle lever (310C-LR, Aurora Scientific) used to prescribe a force profile consisting of 35 linearly spaced forces which start at 0.5 N and end at a maximum of 23 N. The weights of the connecting rod and slider were subtracted from the measurements. The distance was measured at each prescribed force while the device was repeatedly turned turned on and off with a cycle duration of 2 seconds (1 second on). The device was driven with a high-voltage amplifier (TREK 50/12, Advanced Energy) using ramped-edge square waves with amplitudes of 4 kV, 5 kV, or 6 kV and ramp times of 50 ms. To compute the force-stroke curves, we eliminated the first 0.3 s and last 0.2 s of the recorded displacement data for each voltage-on and voltage-off step to eliminate the transient response of the actuator. Then, we took the average of the remaining 0.5 s of displacement data and computed the difference between the voltage-on and voltage-off values to obtain the stroke. The resulting force and stroke curves are shown in (Figure S7). We determined the blocking force of the device as the force at which the device produces a stroke smaller than 1 μm; for 6 kV actuation, the blocking force is around 16 N.

Perceptual Study

The perceptual study was conducted with the TREK 50/12 (Advanced Energy) high-voltage amplifier connected to the safety circuit previously described. The device was attached to the participant's non-dominant wrist using the fabric strap (previously described) and disposable skin-safe adhesive film (2477P, 3M) on the lower surfaces of the conformal spacers; together, these two attachment methods affix the device securely yet comfortably to the user's skin. Throughout the study, participants rested their non-dominant arm on an armrest that did not contact the wrist. They used their dominant hand to directly interact with a Matlab user interface to select a haptic cue to try or to identify the haptic cue they felt. To ensure a complete set of responses during the cue identification task, the data-collection program was set to pause in the event of an electrical breakdown of the actuator, which occurred for only one of the fourteen participants in the study. In this case, the experimenter replaced the actuator with a new one and resumed the test program; no response was collected for the incomplete haptic cue, and the intended cue was automatically repeated.

The forces of the haptic cues used in the perceptual study were measured using a six-axis force-torque sensor (Nano17, ATI Industrial Automation) starting from a non-contact position (approximately 8.7 mm to the base of the actuator). The force sensor was attached to a rigid plate mounted on a height-adjustable adaptor setup. This experiment was conducted using the TREK 10/10B-HS (Advanced Energy).

Supplementary Figures and Tables

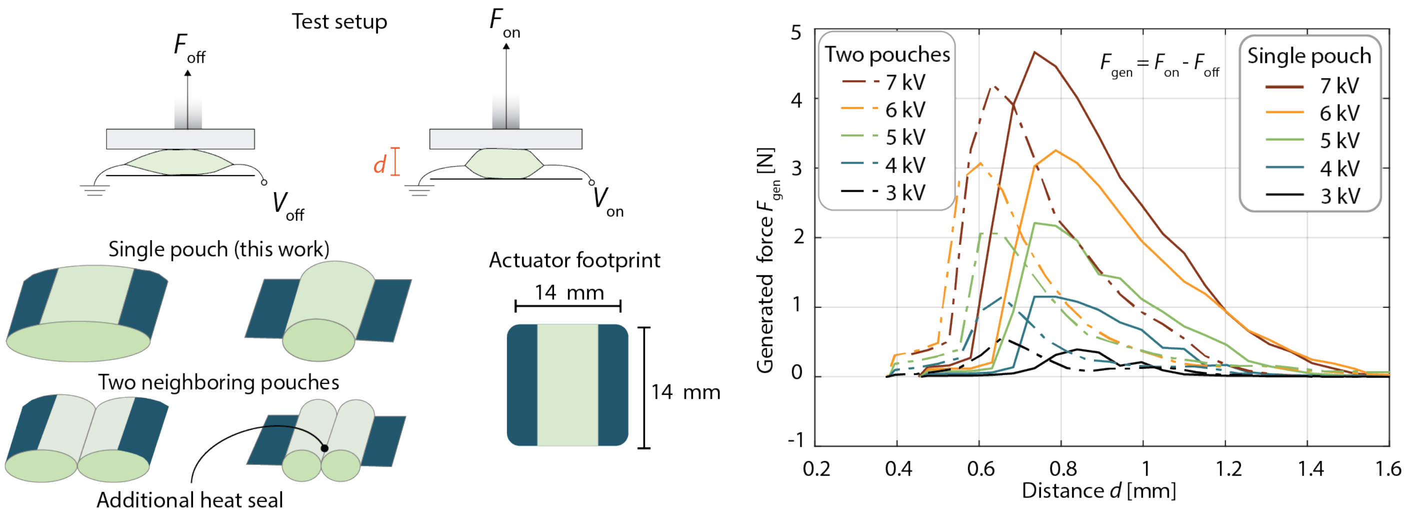

Figure S1: Comparison of the force generated by two designs with the same footprint. The single-pouch design used in this work provides higher forces than two neighboring pouches for actuation voltages above 3 kV. In addition, the single-pouch design can generate forces at a wider range of distances.

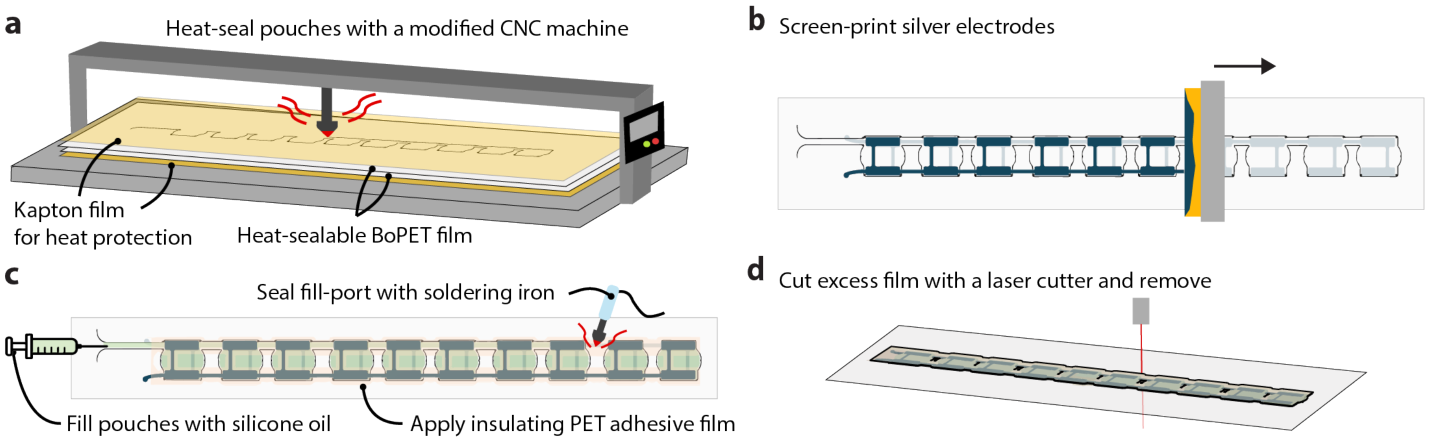

Figure S2: Fabrication process for the electrohydraulic actuators. **a)** Strips of actuator pouches are fabricated by heat-sealing two thermoplastic 15-μm-thick BoPET films. **b)** Electrodes are screen-printed on each side and cured in an oven for 12 minutes. **c)** PET adhesive films are applied over the electrodes on each side of the 10-pouch strip of actuators. Then, the strips are filled with 0.8 ml (0.08 ml per pouch) of a low-viscosity (5 cSt) liquid dielectric (silicone oil). **d)** Each strip of actuators is laser cut to its final shape to remove the mechanical constraints caused by additional film.

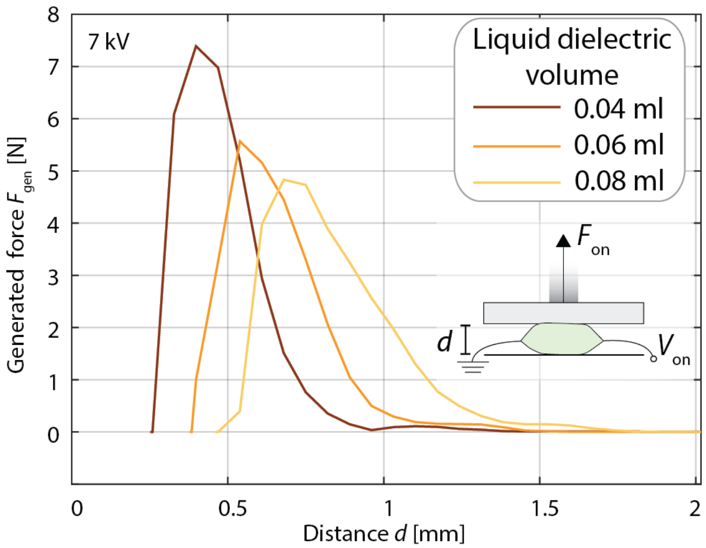

Figure S3: Generated force for three volumes of liquid dielectric oil. There is a trade-off between generated forces and actuator displacement. Larger forces can be generated using a lower volume of liquid dielectric, while higher displacements are achieved with a higher volume. We chose a filling volume of 0.08 ml per pouch to provide forces at a larger range of distances, which facilitates sufficient displacement for making and breaking contact with hairy skin. This parameter can be modified according to the needs of specific applications, e.g., to reach higher forces (over 7 N of force at 7 kV) with less displacement.

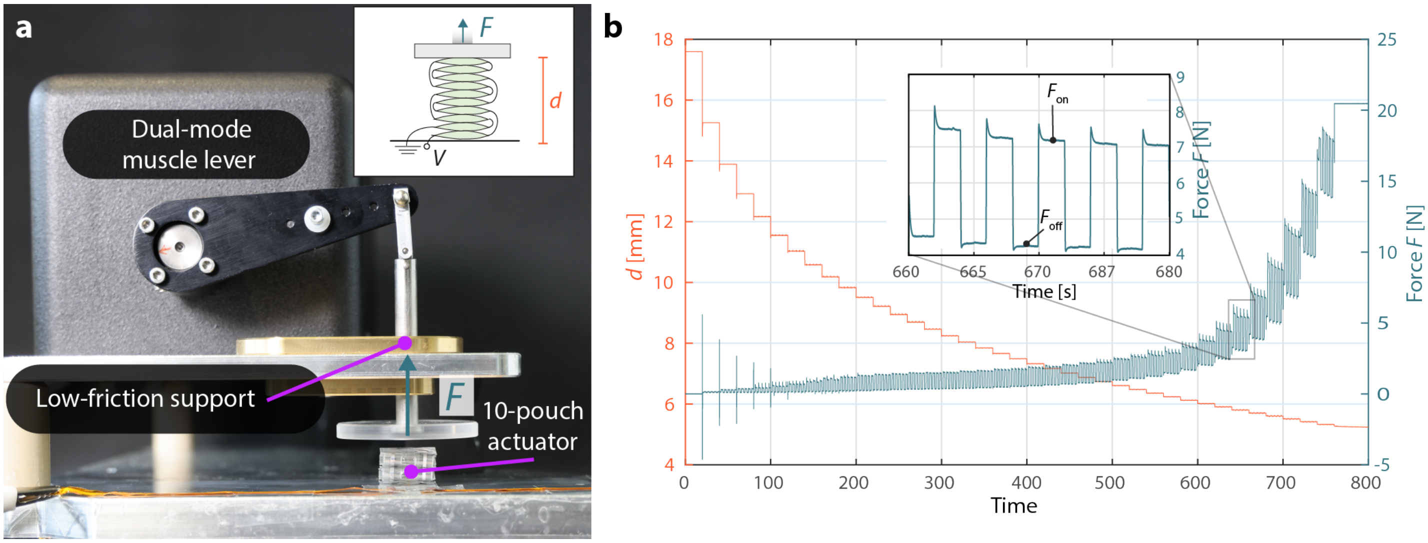

Figure S4: Experimental setup for the measurement of force-distance curves. **a)** A dual-mode muscle lever (310C-LR, Aurora Scientific) was adapted to provide linear motion while measuring displacement and force in compression. **b)** The measured force and prescribed displacement by the muscle lever over 40 distance steps to a maximum force of 20 N. At each step in the depicted experiment, the actuator is turned on (6 kV) and off five times.

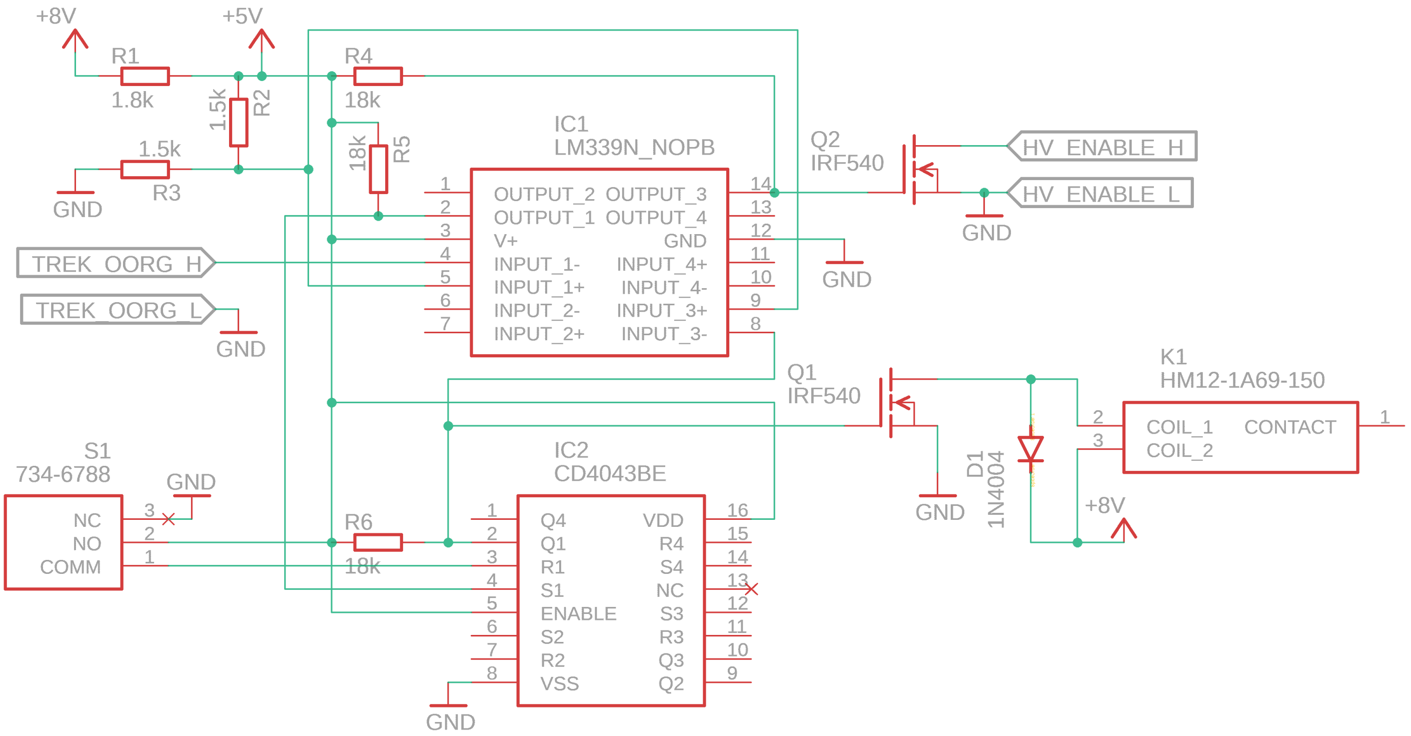

Figure S5: Schematic for the custom safety circuit for automatic shut-off. TREK_OORG is the out-of-regulation status output of the high-voltage amplifier. HV_ENABLE is an input of some high-voltage amplifier models (e.g., Trek 50/12) that enables high-voltage output. The high-voltage output is deactivated when the HV_ENABLE is disconnected from ground. Thus, when the OORG status becomes low (0 V), the circuit disables high voltage by disconnecting HV_ENABLE from ground.

Part	Part Number	Description
D1	1N4004	Diode
IC1	LM_339N_NOPB	Comparator
IC2	CD4043BE	SR latch
K1	HM12-1A69-150	High-voltage relay
Q1-2	IRF540	N-channel MOSFET
R1		1.8 kΩ resistor
R2, R3		1.5 kΩ resistor
R4-R6		18 kΩ resistor
S1	734-6788 (RS Components)	SPDT push-button switch

Table S1: List of parts for the custom safety ciruit.

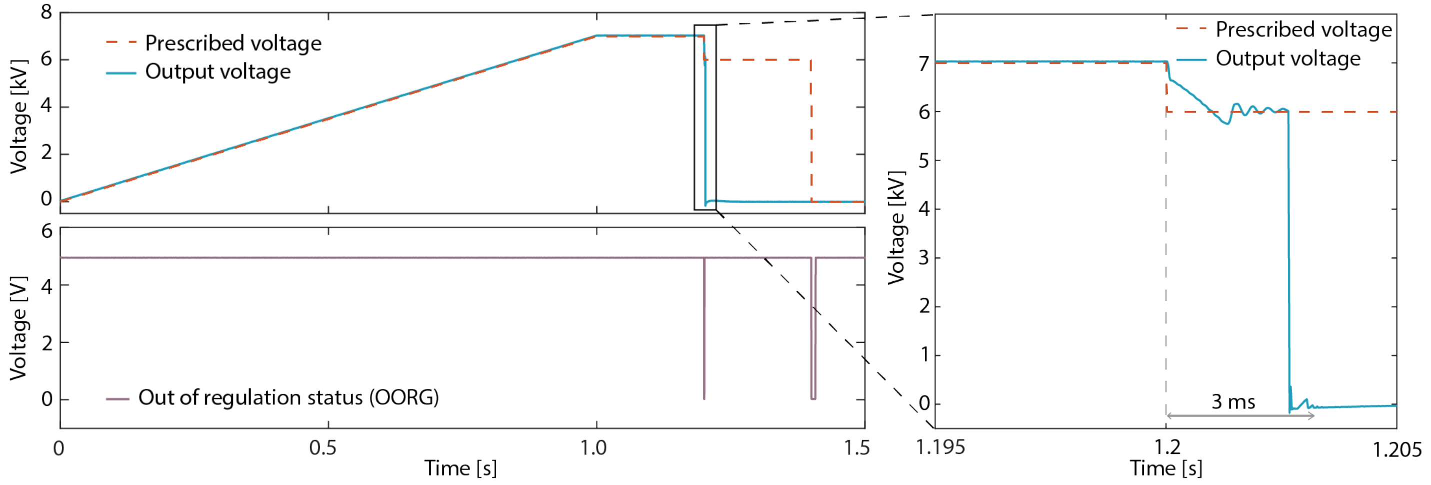

Figure S6: Measurement of automatic shut-off time. We applied an actuation waveform that ramps up the voltage to 7 kV, holds the actuator at this voltage for 1 second, and then rapidly reduces the voltage to 6 kV. The fast discharge (0.02 ms) triggers the OORG status, mimicking an event such as an electrical breakdown. We chose this driving signal to show the combined time for the activation of the OORG status and the time it takes to discharge the actuator from 6 kV. This test shows that the safety circuit disables high voltage and discharges the actuator in less than 3 ms.

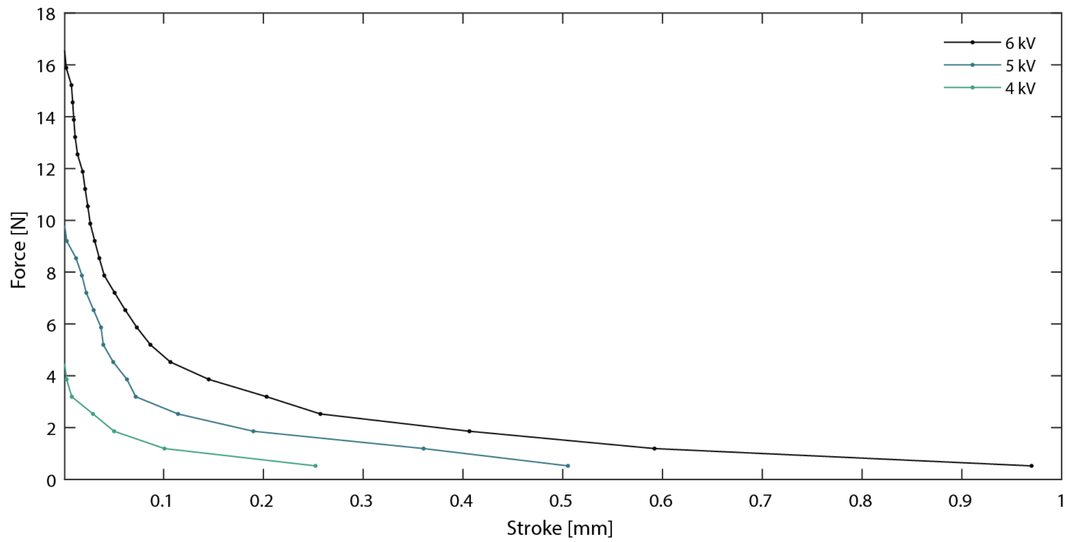

Figure S7: Stroke (peak-to-peak displacement) and applied force of the device at actuation voltages of 4 kV, 5 kV, and 6 kV. For 6 kV actuation, the device has a blocking force of approximately 16 N (i.e., the force at which the actuator has a stroke than 1 μm). For this measurement, we employed a dual-mode muscle lever (310C-LR, Aurora Scientific), which provided 35 steps of applied force (up to 23 N) while measuring displacement and force in compression.

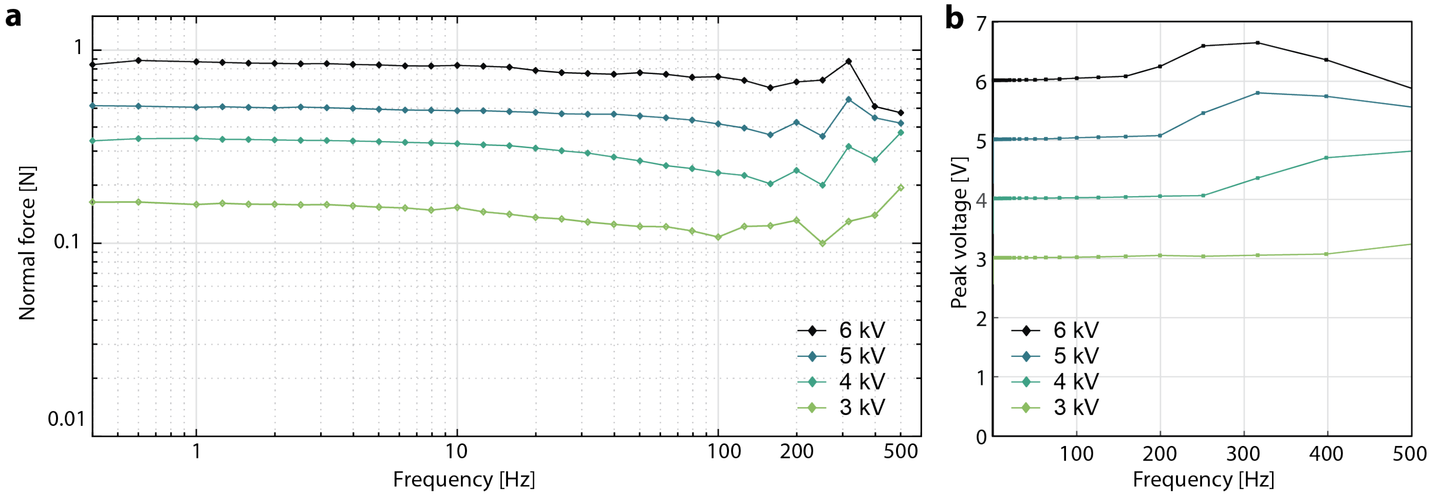

Figure S8: **a)** Peak-to-peak output force for sinusoidal input signals from 0.4 Hz to 500 Hz in octaves, for a distance $d = 8.5$ mm and peak voltages from 3 kV to 6 kV. The high-voltage amplifier TREK 610E (Advanced Energy) was used for this test, and each frequency was measured over 5 s. **b)** Peak measured voltage. We observed that the peak voltage partially deviated from the prescribed value for higher frequencies; this effect became substantial for frequencies above 200 Hz and larger peak-to-peak voltages.

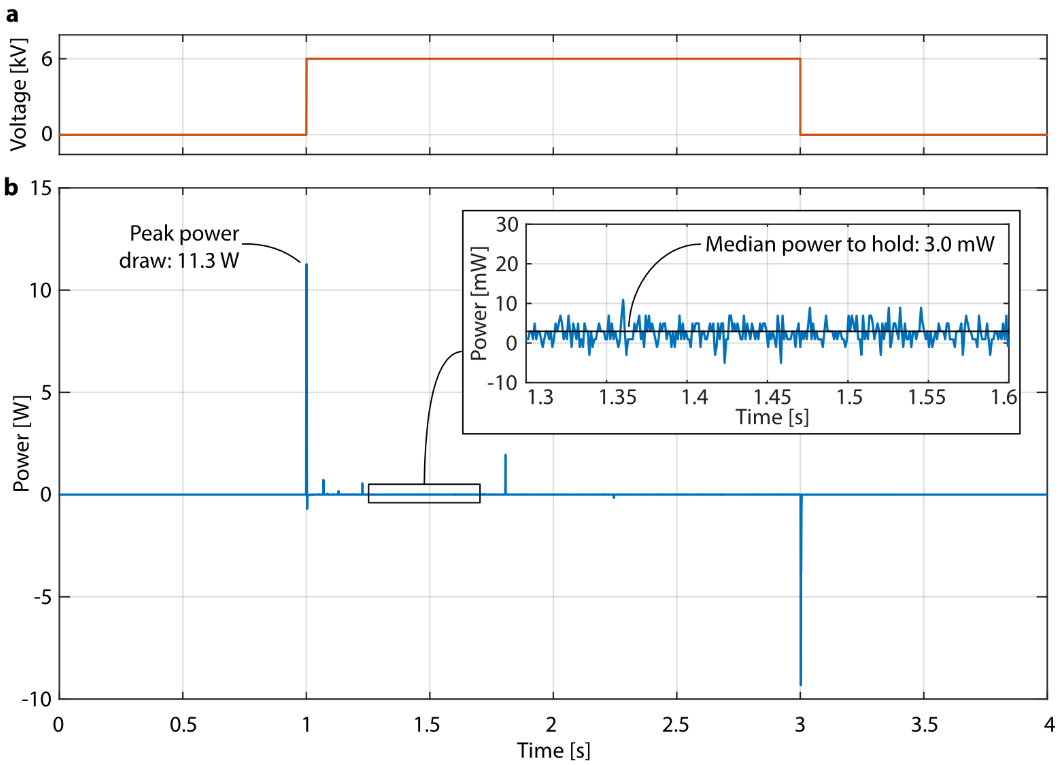

Figure S9: Measurement of power consumption of the CUTE device. **a)** Driving voltage signal used for this experiment. **b)** Measured power drawn by the CUTE device. We used a high-voltage amplifier (TREK 50/12, Advanced Energy) to apply and record the voltage and used an electrometer (Model 6514, Keithley) to measure the current on the ground side of the device. We multiplied voltage and current to compute electrical power.

Signal	Voltage waveform	Participant responses
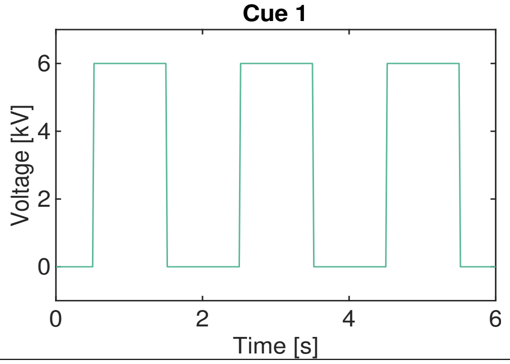	Square wave, 0.5 Hz (1 second on, 1 second off)	**Description:** tapping (5), pressing (3), pulse/beat (2) **Metaphors:** clock (4), someone or something tapping (4), something drawing attention (2), heartbeat (2) **Suggested uses:** notification or reminder (6), convey time or tempo (4), gaining attention (2)
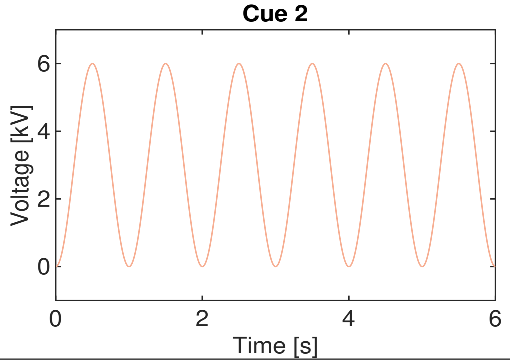	Sine wave, 1 Hz (0–6 kV)	**Description:** life-like (3), soft or mild (3), something rolling (2), slow (2) **Metaphors:** water flowing, ocean or rhythmic waves (4), heartbeat (4), life-like (3), breathing (2), massage (2), pulsing (2) **Suggested uses:** light alarm (4), simulate the breath or pulse of a living thing (3)
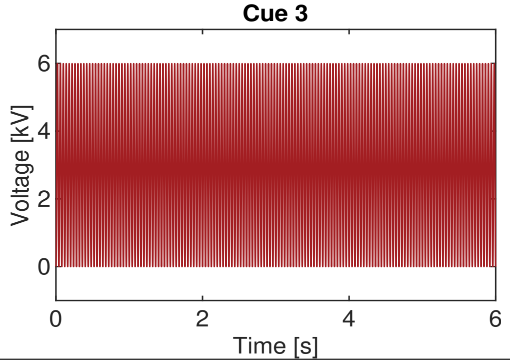	Sine wave, 25 Hz (0–6 kV)	**Description:** vibration (8), urgent (2) **Metaphors:** vibration (5), alarm (4) **Suggested uses:** alarm or notification (8), indicate proximity (4), alert about dangers or dangerous situations (3), warning signal (2)
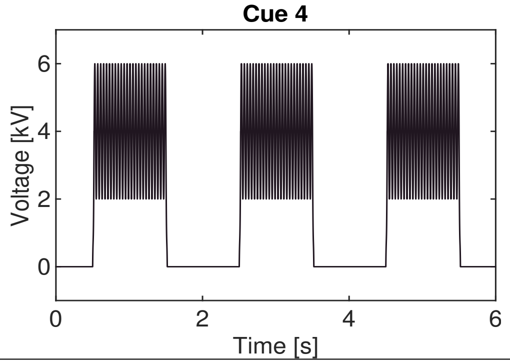	Pulsed sine wave, 25 Hz (2–6 kV) (1 second on, 1 second off)	**Description:** vibration (6), pause (3), not urgent (2), alarm (2) **Metaphors:** phone or telephone (9), vibration (4) **Suggested uses:** notification or reminder (8), alarm (5), calling attention (4)

Table S2: Definitions of signals 1–4 used in the perceptual study, along with common participant descriptions; the numbers in parentheses indicate the number of participants who mentioned a version of each listed response.

Signal	Voltage waveform	Participant responses
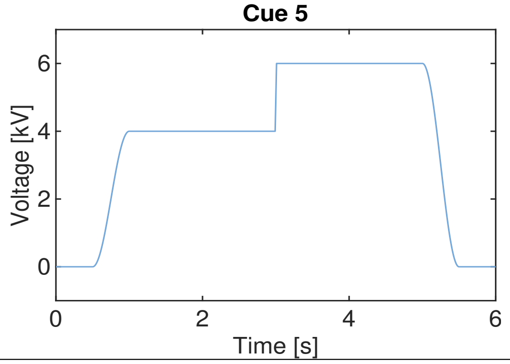	2 steps (4.5 kV, 6 kV) with a square step from 4.5 to 6 kV and half sine wave (1 Hz) transitions at the beginning and the end	**Description:** touch (5), tap (2), push (2)<br>**Metaphors:** Two phase sensation of pressure (2)<br>**Suggested uses:** positive feedback (2), communicate something not urgent (2), guidance (2)
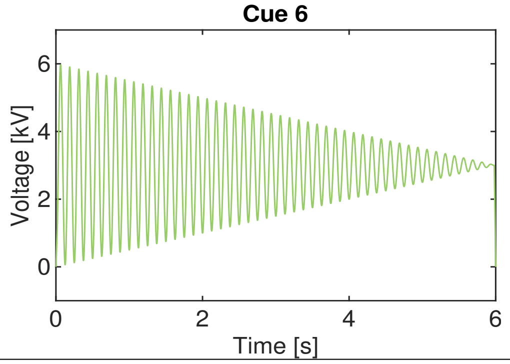	Sine wave, 8 Hz from 6 kV to 0 kV amplitude vibration with decaying amplitude. The signal is centered around 3 kV.	**Description:** vibration (5), fading out (3)<br>**Metaphors:** vibration (3), something stopping or turning off (3)<br>**Suggested uses:** something finishing or ending (3), proximity sensing or guidance (2)
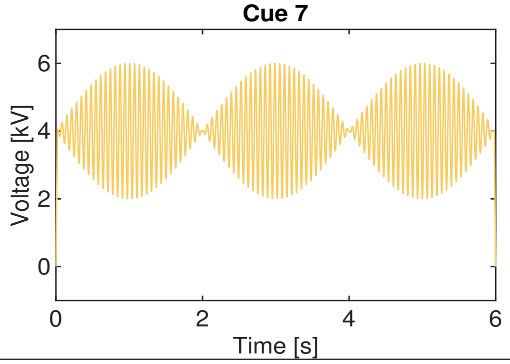	3 pulses of a sine wave, 15 Hz (2 kV amplitude), with a 4 kV bias	**Description:** vibration (6)<br>**Metaphors:** phone (3), life-like i.e. wings, cat sounds (3), vehicle accelerating/decelerating or motor starting (2)<br>**Suggested uses:** calming (2), not urgent notification (2), light alarm (2), timer (2)
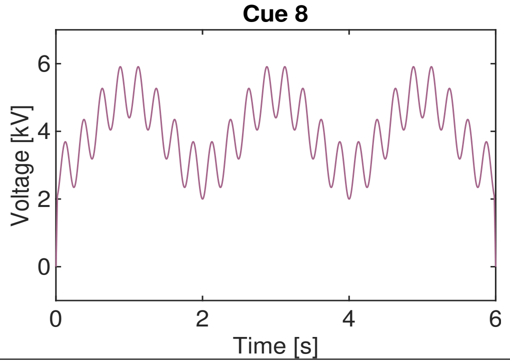	4 Hz vibration (0.8 kV amplitude) + 0.5 Hz actuation (1.2 kV amplitude) with a 4 kV bias	**Description:** light (3), waves (2), pressing/pressure (2), steps or staircase (2)<br>**Metaphors:** stroking or tickling on the skin (3), heart (2)<br>**Suggested uses:** notification (3), knob (2)

Table S3: Definitions of signals 5–8 used in the perceptual study, along with common participant descriptions; the numbers in parentheses indicate the number of participants who mentioned a version of each listed response.

Signal	Voltage waveform	Participant responses
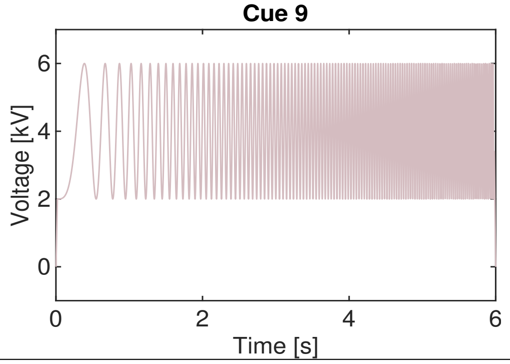	Linearly increasing chirp from 0 to 40 Hz (2–6 kV).	**Description:** vibration (7), increasing (5)<br>**Metaphors:** start of an engine, motor, car, or mechanical system (9)<br>**Suggested uses:** something starting/turning on (5), indicate proximity (3), notification (3), acceleration (2)
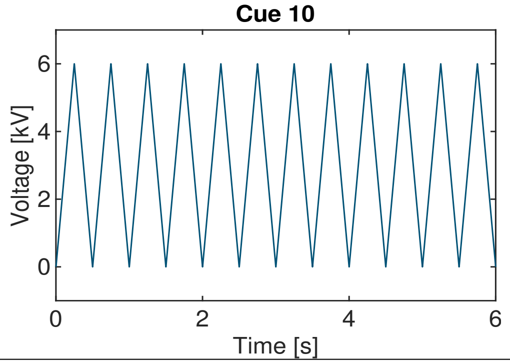	Triangular wave, 2 Hz, (0–6 kV)	**Description:** similar to sinusoidal wave (cue 2) (3) but faster (2), fast (3)<br>**Metaphors:** heartbeat (4), small (2)<br>**Suggested uses:** alarm, alert, notification (4), reminder (2)
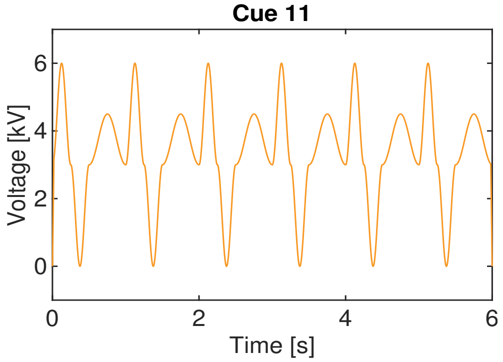	Periodic signal repeating at 1 Hz, two high peaks of 4.5 kV and 6 kV, and two troughs of 0 kV and 3 kV	**Description:** lifelike, like a heart or muscle (3), similar to triangular (cue 10) (2)<br>**Metaphors:** heartbeat (4), someone pushing lightly, poking gently, or touching (3), like animals, i.e. horse galloping, worms, heartbeat of a small animal (3)<br>**Suggested uses:** waiting for something in progress (3), medical test or health monitor (3)

Table S4: Definitions of signals 9–11 used in the perceptual study, along with common participant descriptions; the numbers in parentheses indicate the number of participants who mentioned a version of each listed response.

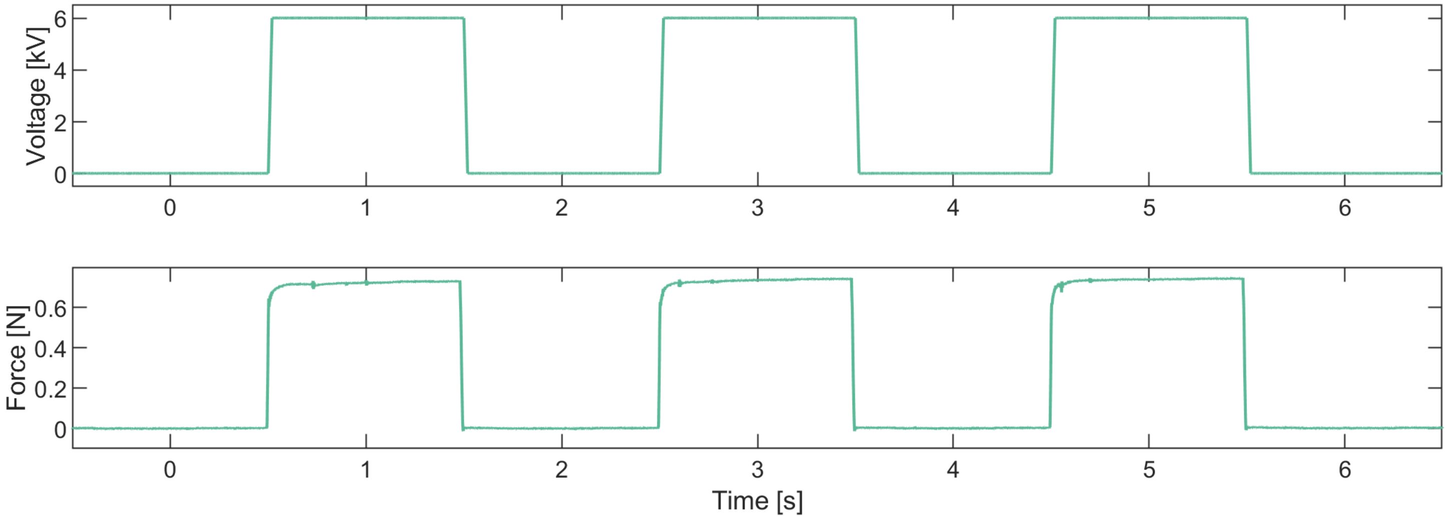

Figure S10: Expanded view of haptic cue 1.

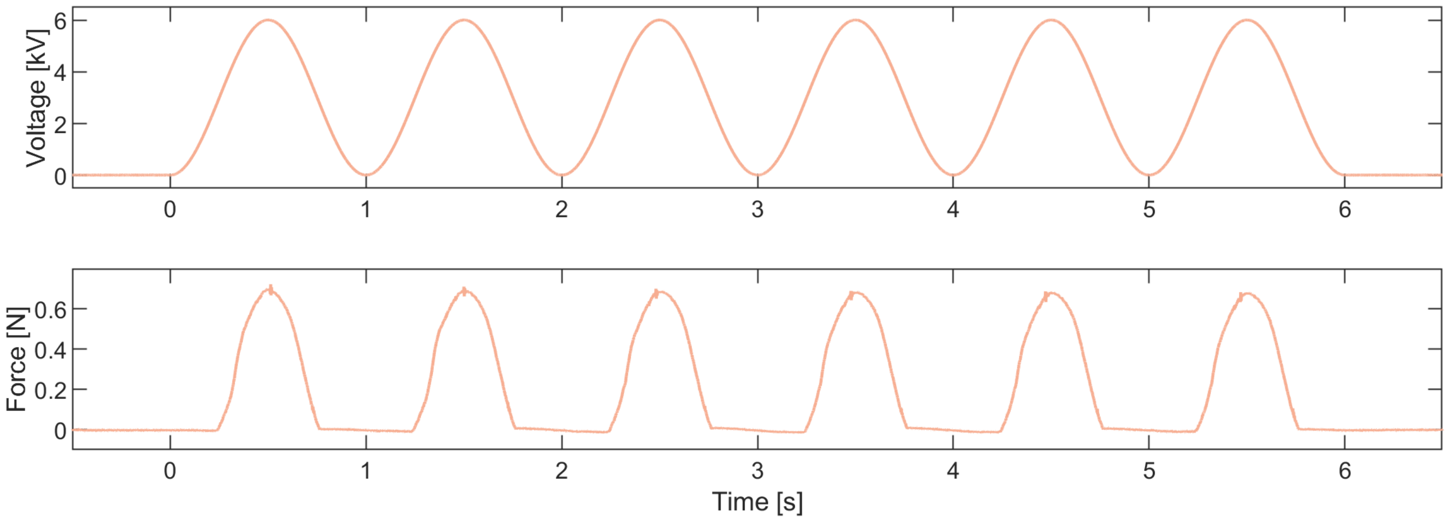

Figure S11: Expanded view of haptic cue 2.

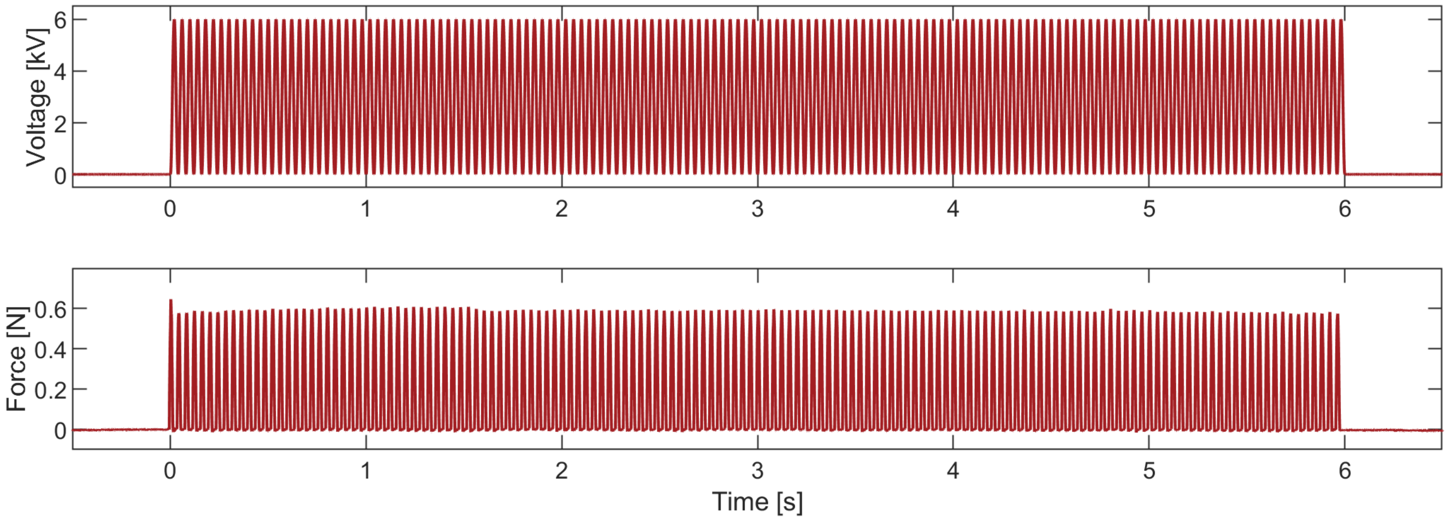

Figure S12: Expanded view of haptic cue 3.

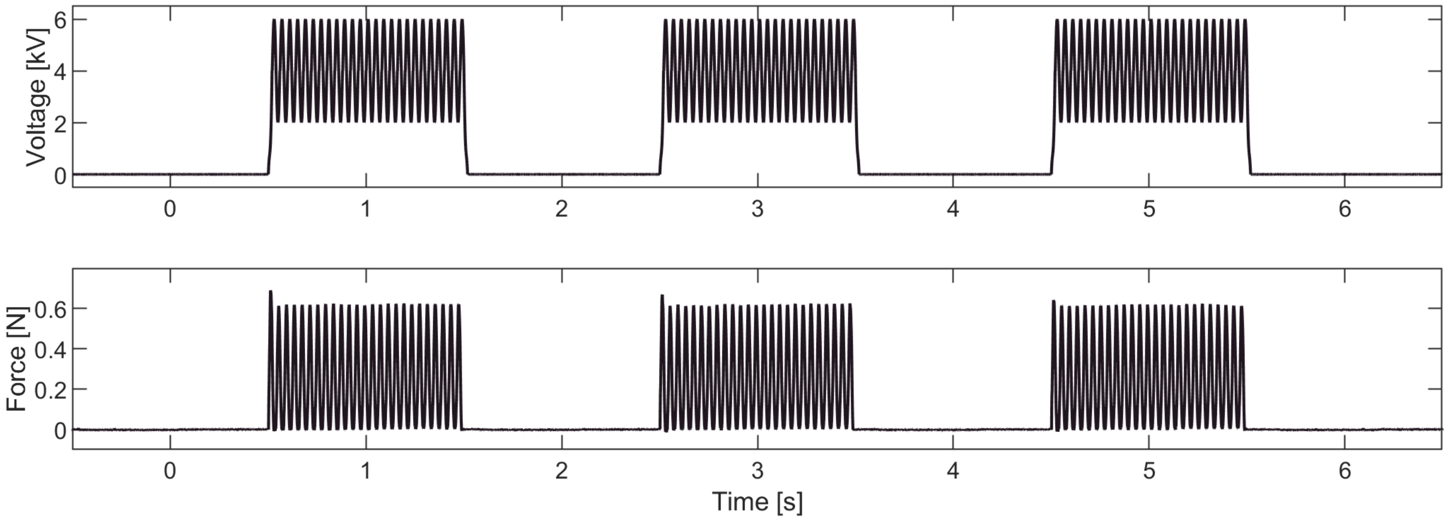

Figure S13: Expanded view of haptic cue 4.

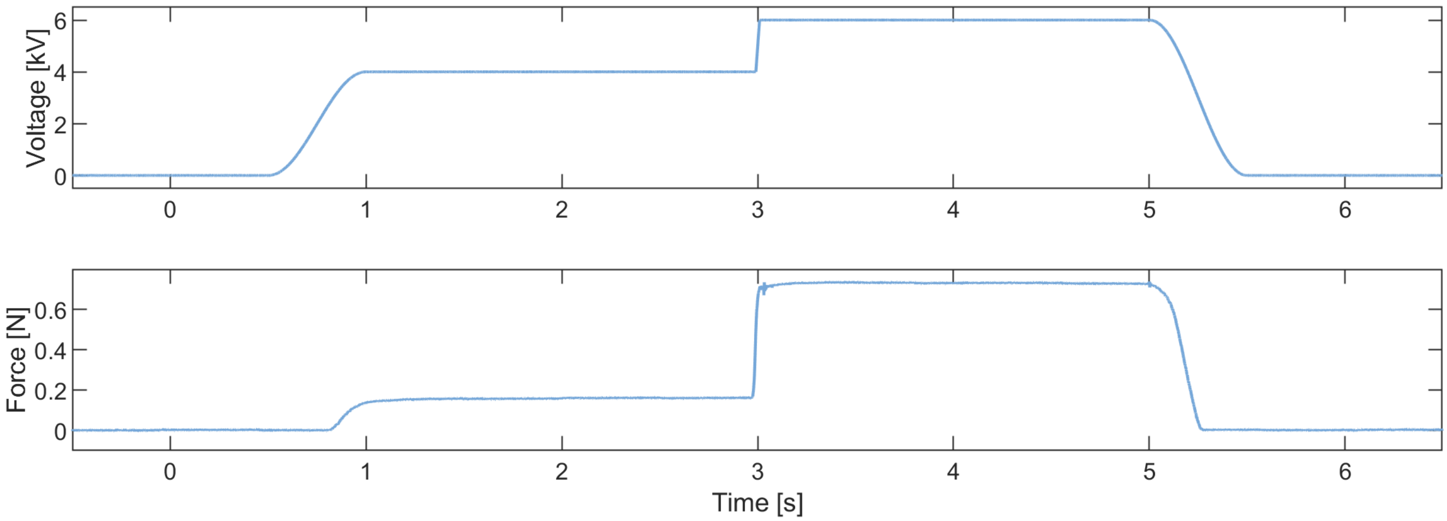

Figure S14: Expanded view of haptic cue 5.

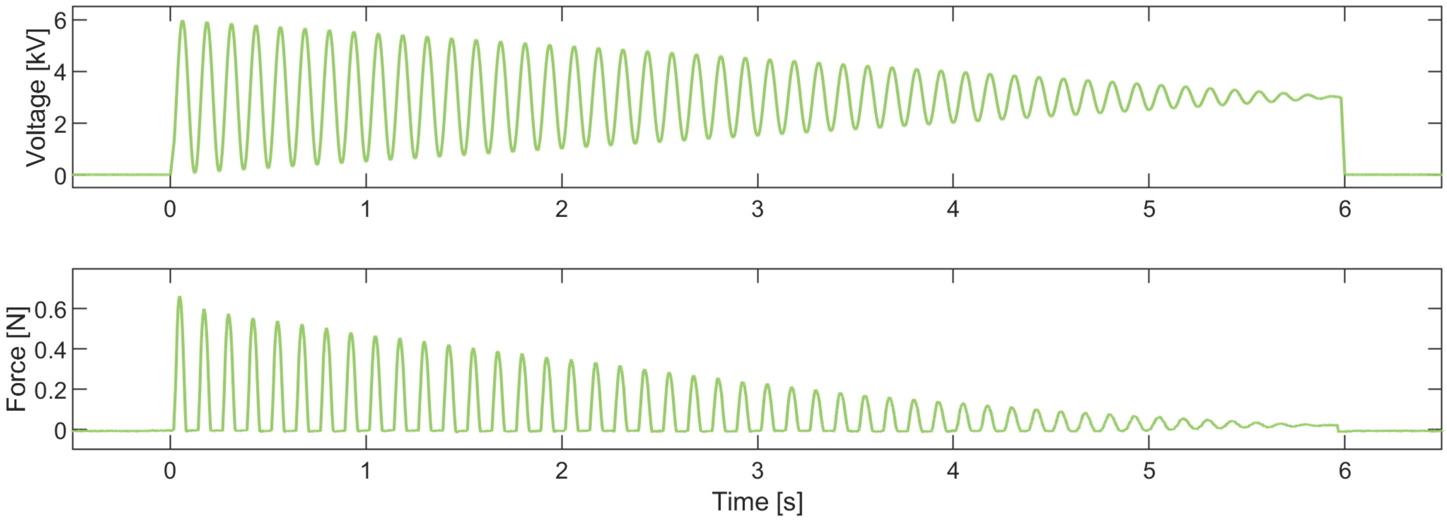

Figure S15: Expanded view of haptic cue 6.

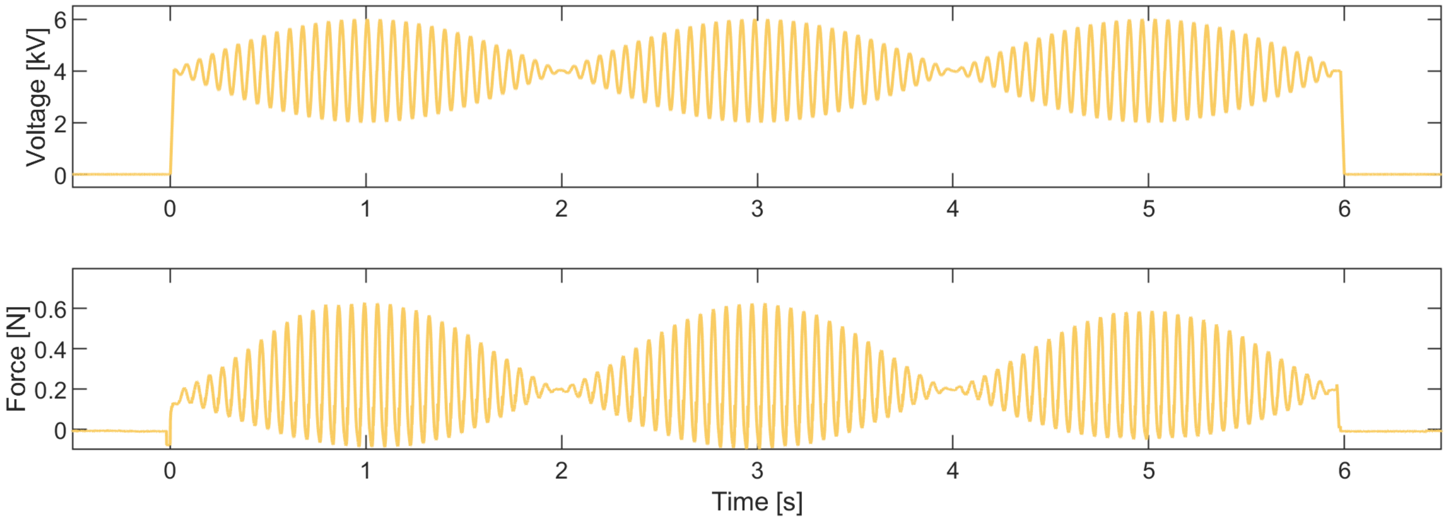

Figure S16: Expanded view of haptic cue 7.

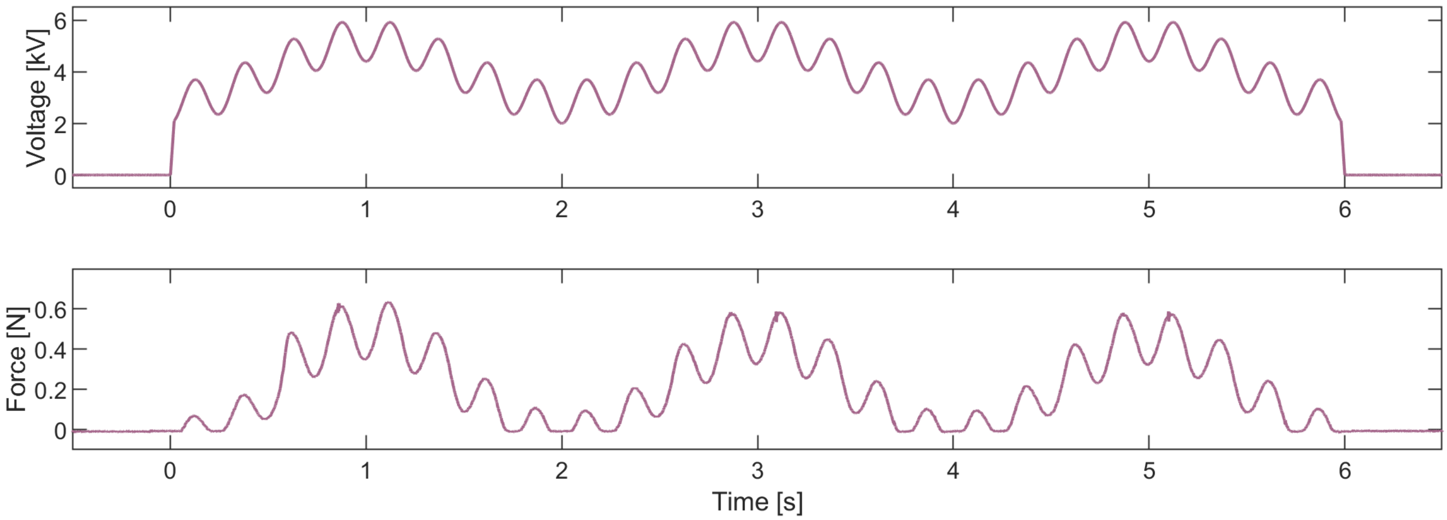

Figure S17: Expanded view of haptic cue 8.

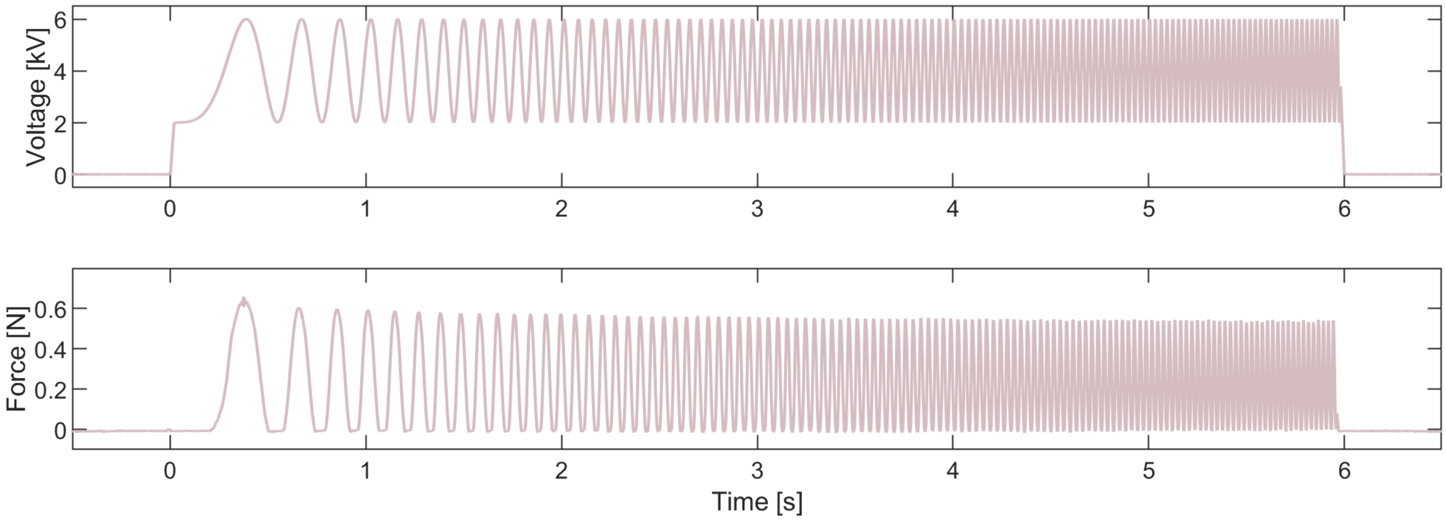

Figure S18: Expanded view of haptic cue 9.

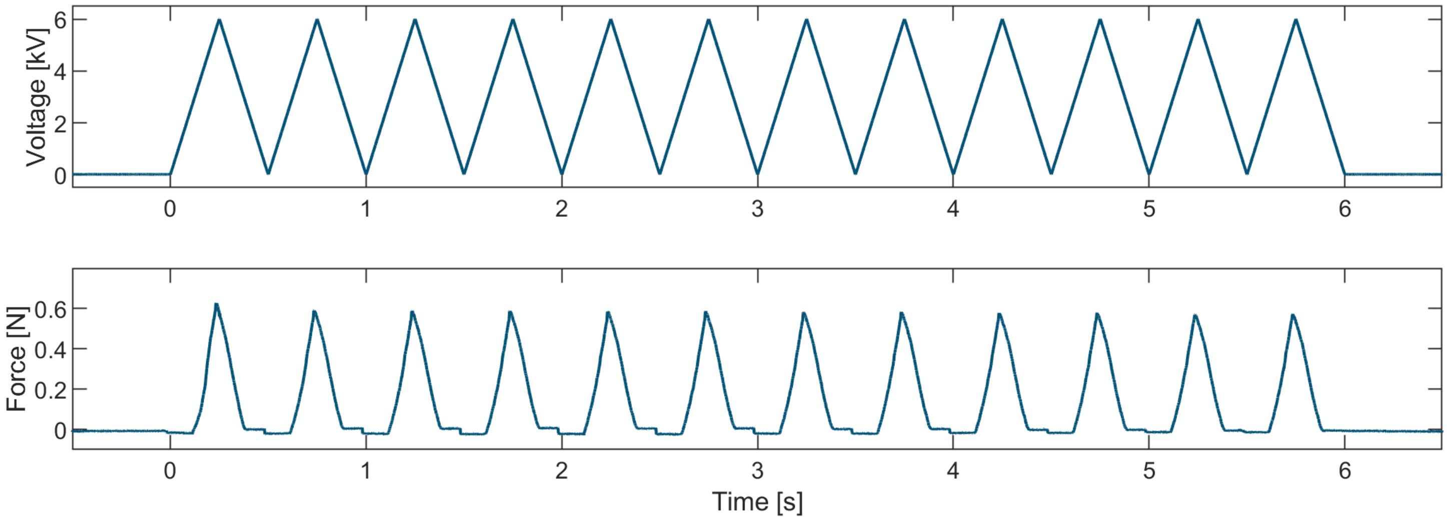

Figure S19: Expanded view of haptic cue 10.

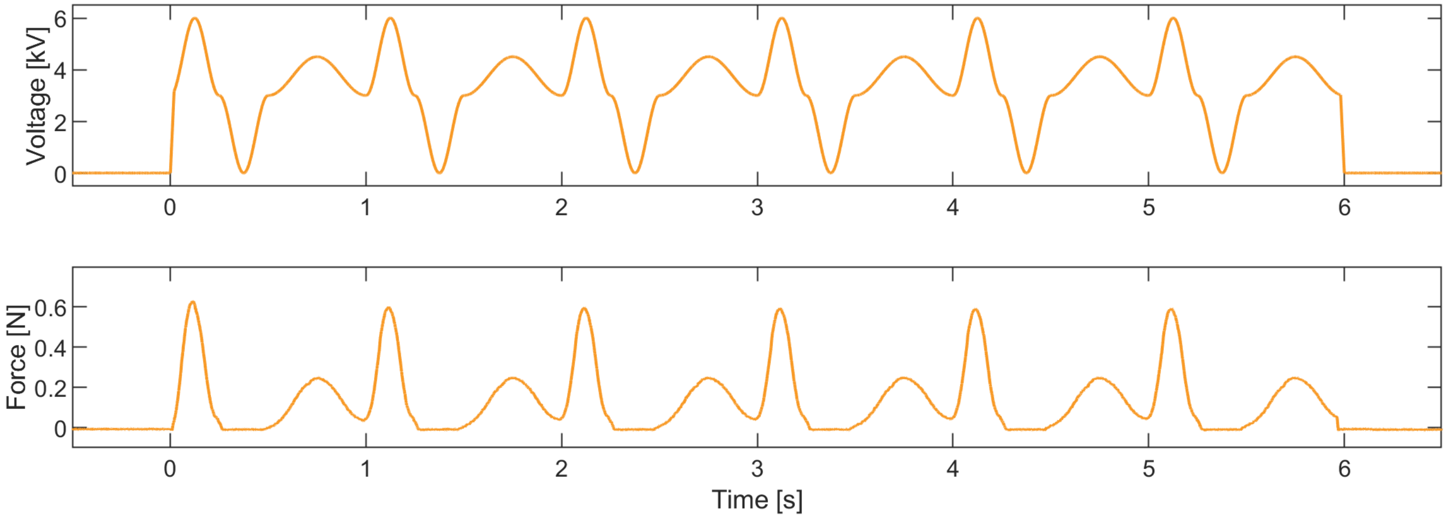

Figure S20: Expanded view of haptic cue 11.

Supplementary Movies

Movie S1: Key features of the cutaneous electrohydraulic (CUTE) wearable device. The device is worn on the wrist, and diverse haptic cues are demonstrated, including multiple actuation patterns, at different amplitudes and frequencies. The actuator's surface is visibly coming into and out of contact with the skin.

Movie S2: CUTE devices do not heat up during actuation. The temperature of a CUTE device and a voice-coil actuator were measured during continuous low-frequency actuation. The voice-coil actuator heats up from room temperature to 42 °C in four minutes of 10 Hz actuation, while the CUTE device remains at room temperature.

Movie S3: A CUTE device demonstrates the five haptic cues used in the cue identification task. The cues are shown both in free space and actuated on the skin, with a high-speed video of cues 3 and 4, which display vibration at 25 Hz.

Movie S4: The six additional haptic cues that the CUTE device presented to participants during the cue description task. Videos of the CUTE device actuated in free space and on the skin are displayed, including a high-speed video of cue 9, which provides vibrations up to 40 Hz.

References

[1] S. K. Mitchell, X. Wang, E. Acome, T. Martin, K. Ly, N. Kellaris, V. G. Venkata, C. Keplinger, *Advanced Science* **2019**, *6*, 1900178.
[2] X. Wang, S. K. Mitchell, E. H. Rumley, P. Rothemund, C. Keplinger, *Advanced Functional Materials* **2020**, *30*, 1908821.
[3] T. Wang, H.-J. Joo, S. Song, W. Hu, C. Keplinger, M. Sitti, *Science Advances* **2023**, *9*, eadg0292.
[4] N. Kellaris, V. G. Venkata, P. Rothemund, C. Keplinger, *Extreme Mechanics Letters* **2019**, *29*, 100449.
[5] Standard for electrical safety in the workplace, Standard, National Fire Protection Association (NFPA), **2024**.
[6] Effects of current on human beings and livestock, Standard, International Electrotechnical Commission (IEC), **2018**.
